# Supplementary material for: Barriers to and facilitators of the identification, management and referral of childhood anxiety disorders in primary care: a survey of general practitioners in England
Source: BMJ Open. 2019 Apr 23;9(4):e023876. doi: 10.1136/bmjopen-2018-023876 (PMC6501977; doi:10.1136/bmjopen-2018-023876)
Supplement: Supplementary data [file bmjopen-2018-023876supp001.pdf]

CADPC

Consent page

Children with Anxiety Disorders in Primary Care: the views of General Practitioners II

We invite you to take part in a National Institute of Health Research funded study which focuses on management of childhood anxiety disorders (children under 12 years) by General Practitioners in England. This study explores GPs' experiences of identification, management and referral of these disorders.

Please note, while some of these questions may seem repetitive, consider whether they are being asked in relation to either identification, management or referral. Your responses will help inform practice guidelines for children with anxiety disorders.

Taking part in this study will involve completing a survey, which should take 10-15 minutes to complete. This is open to as many GPs per surgery as desired.

As a reimbursement for taking part in the study you (or a nominated member of your practice) will have the opportunity to take part in a Webinar about identification and management of childhood anxiety disorders. This module may be used to contribute towards Continued Professional Development points and a certificate of attendance outlining your involvement in important mental health research will be provided. You will also be entered into a prize draw to win one of two £100 Amazon vouchers.

We are happy to answer queries about the study, please contact the researchers for further information:

Doireann O'Brien, B.A., M.Sc.
Email: doireann.obrien@pgr.reading.ac.uk

Professor Cathy Creswell
Tel: +44 (0)118 378 6798

Further details of the study are available as an information sheet. Please find information sheet enclosed.

All items of the consent form, below, must be initialled to indicate consent.

I can confirm that I have read and understand the information sheet, have had the opportunity to ask questions and, if applicable, have had these questions answered satisfactorily ⓘ *Required* ☐

I understand that my participation is voluntary, that I can withdraw my consent at any time, without giving a reason and without my professional role being compromised ⓘ *Required* ☐

I understand that the answers to my questions will be stored in a secure location, but will bear no identifying information. I also understand that the data will be kept for 12 months and after this time they will be destroyed ⓘ *Required* ☐

I understand that, should I lose the capacity to consent to be in the study, the research centre will retain any information collected prior to this point ⓘ *Required* ☐

I understand that relevant sections of my data collected during the study, may be looked at by individuals from the University of Reading, from regulatory authorities or from the NHS Trust, where it is relevant to my taking part in this research. I give permission for these individuals to have access to my records ⓘ *Required* ☐

I agree to take part in the study ⓘ *Required* ☐

SURVEY

Age: *(please circle)* 23-35 35-45 45-55 55-65 65+
Identify my gender as: *(please circle)* MALE FEMALE OTHER
Psychiatric rotation completed as part of GP training: *(please circle)* YES NO
Paediatric rotation completed as part of GP training: *(please circle)* YES NO
Year qualified as a GP:
Practice Name:
Practice Postcode:
Years employed in current Trust:
Would you consider yourself research active? *(please circle)* YES NO
Are you a parent or guardian? *(please circle)* YES NO
If so, what ages are your children:
Compared to other GPs in your practice, do you see more or fewer child patients? *(please circle)* YES NO SIMILAR AMOUNT

PLEASE NOTE: **ALL QUESTIONS ARE IN RELATION TO ANXIETY IN CHILDREN AGED 12 & UNDER (i.e. primary school age and below)**

For each item identified below, circle the number to the right that best fits your judgment of its quality.
Use the rating scale to select the number.

**Survey Item**	**Scale***<br>**1 = completely disagree**<br>**2 = disagree**<br>**3 = neutral**<br>**5 = agree**<br>**5 = completely agree**<br><br>*unless otherwise specified				
**Identification**<br>*These questions are in relation to initially identifying the presence of an Anxiety Disorder in a child*					
1. How common are anxiety disorders in children in your experience?	1 in 3	1 in 10	1 in 15	1 in 20	1 in 40
2. How often do you see anxiety disorders in your practice?					
3. I believe that anxiety disorders are prevalent in children under 12	1	2	3	4	5
4. I am confident in my ability to recognise anxiety disorders in children	1	2	3	4	5
5. I am regularly looking out for anxiety disorders in children	1	2	3	4	5
6. My training adequately equipped me with the skills to identify anxiety disorders in children	1	2	3	4	5
7. I use screening tools to aid identification of anxiety disorders in children (Screening tools refer to validated measures used to aid identification of a suspected anxiety disorder)	Yes			No	
a. *If yes:* I find screening tools useful for the identification of anxiety disorders in children	1	2	3	4	5
b. *If no:* I would find a brief screening tool useful for identification of anxiety disorders in children	1	2	3	4	5
8. I am comfortable broaching the subject of a potential anxiety disorder with:					
a. the child	1	2	3	4	5
b. the family	1	2	3	4	5
9. I believe that it is part of my responsibility in my role as a GP to identify anxiety disorders in children	1	2	3	4	5
10. Other agencies are responsible for identifying childhood anxiety disorders	1	2	3	4	5
a. *If yes*: Name agencies here					

11. To what extent do the following factors get in the way of your ability to accurately identify anxiety disorders in children:	**For the following:**<br>**1 = Not at all**<br>**2= A little**<br>**3 = Somewhat**<br>**4 = Quite a lot**<br>**5 = Very much**				
a. Limitations in children's communication abilities	1	2	3	4	5
b. Misinformation from parents	1	2	3	4	5
c. My concerns about stigmatising the child	1	2	3	4	5
d. Family concerns about stigma	1	2	3	4	5
e. Cultural barriers	1	2	3	4	5
f. Language barriers	1	2	3	4	5
g. Time restrictions	1	2	3	4	5
h. Family reluctance to accept disorder	1	2	3	4	5
i. Lack of training	1	2	3	4	5
j. Lack of experience	1	2	3	4	5
k. Lack of knowledge	1	2	3	4	5
l. Lack of available/accessible treatment	1	2	3	4	5
m. Lack of effective treatment	1	2	3	4	5
Please add any further barriers to identification here if necessary					
n.	1	2	3	4	5
o.	1	2	3	4	5
p.	1	2	3	4	5
q.					
r.					

**Survey Item**	**Scale***<br>**1 = completely disagree**<br>**2 = disagree**<br>**3 = neutral**<br>**5 = agree**<br>**5 = completely agree**<br><br>*unless otherwise specified

Management:

These questions are in relation to your ongoing management of a childhood Anxiety Disorders, either prior to making a referral or in the absence of a referral

12. I believe that I have had adequate training to manage anxiety disorders in children	1	2	3	4	5
13. I have adequate skills to manage anxiety disorders in children	1	2	3	4	5
14. I feel confident managing anxiety disorders in children	1	2	3	4	5
15. I am comfortable discussing management strategies for anxiety disorders with:					
a. the child	1	2	3	4	5
b. the family	1	2	3	4	5
16. I provide families with management tips & strategies for managing anxiety disorders in children	1	2	3	4	5
17. I am aware of resources such as books/websites/apps to aid families in the management of anxiety disorders in children	Yes			No	
a. *If yes:* I use resources such as books/websites/apps in the management of childhood anxiety disorders	1	2	3	4	5
b. *If yes:* Please state which ones					
c. *If no:* I would use resources such as books/websites/apps in the management of childhood anxiety disorders	1	2	3	4	5
18. Are you aware of any online/apps resources for parents concerned about their child's anxiety?	YES			NO	
a. *If Yes*: please state which ones					
b. *If Yes:* I have sign-posted parents concerned about their child's anxiety to these	1	2	3	4	5
c. *If No:* If I were aware of online/app resources, I would signpost parents to these if they were concerned about their child's anxiety	1	2	3	4	5
19. It would be important for an online/app resource for childhood anxiety to have an evidence-base in order for me to recommend it	1	2	3	4	5
20. It would be important to have NHS accreditation of the online/resources for childhood anxiety be in order for me to recommend it	1	2	3	4	5
21. Having a relationship with a family aids me in the management of anxiety disorders	1	2	3	4	5
22. I involve the child's school in the management of anxiety disorders	1	2	3	4	5
a. *If yes:* I find this helpful	1	2	3	4	5
23. I am aware of local agencies available to support children with anxiety disorders and their families	1	2	3	4	5
24. I believe it is part of my role as a GP to manage anxiety disorders in children	1	2	3	4	5
25. Other agencies are responsible for managing childhood anxiety disorders	1	2	3	4	5
a. Add other agencies here if applicable					

26. To what extent do the following factors get in the way of your ability to effectively manage anxiety disorders in children:	**For the following:**<br>**1 = Not at all**<br>**2= A little**<br>**3 = Somewhat**<br>**4 = Quite a lot**<br>**5 = Very much**				
a. Cultural barriers	1	2	3	4	5
b. Language barriers	1	2	3	4	5
c. Time restrictions	1	2	3	4	5
d. Family reluctance to accept the disorder	1	2	3	4	5
e. Limitations in children's communication abilities	1	2	3	4	5
f. Misinformation from parents	1	2	3	4	5
g. My concerns about stigmatising the child	1	2	3	4	5
h. Family concerns about stigma	1	2	3	4	5
i. Lack of training	1	2	3	4	5
j. Lack of experience	1	2	3	4	5
k. Lack of knowledge	1	2	3	4	5
Please add any further barriers to management below if necessary					
l.	1	2	3	4	5
m.	1	2	3	4	5
n.	1	2	3	4	5

**Survey Item**	**Scale***<br>**1 = completely disagree**<br>**2 = disagree**<br>**3 = neutral**<br>**5 = agree**<br>**5 = completely agree**<br><br>*unless otherwise specified				
**Referral**<br>*These questions are in relation to a referral of a childhood Anxiety Disorder to a specialist service (such as Child & Adolescent Mental Health Services)*					
27. How often in the last 5 years have you referred a child to specialist services for an anxiety disorder?	0 times	0-5 times	5-10 times	10-15 times	15+ times
28. Parental pressure increases the likelihood that I would refer a child with an anxiety disorder to specialist services	1	2	3	4	5
29. anxiety disorders in children will generally resolve itself without treatment	1	2	3	4	5
30. I seek specialist advice prior to making a referral of a childhood anxiety disorder	1	2	3	4	5

a. *If yes:* I find this helpful	1	2	3	4	5
b. *If no:* I think that I would find this helpful	1	2	3	4	5
31. I experience long waiting times for referral for a childhood anxiety disorders	1	2	3	4	5
a. *If yes:* Long waiting times reduce the likelihood that I would make a referral to specialist services for childhood anxiety disorders	1	2	3	4	5
32. I feel like I have a relationship with specialist services for childhood anxiety disorders	1	2	3	4	5
a. *If yes:* This is helpful when making a referral	1	2	3	4	5
b. *If no:* I think this would this be helpful when making a referral	1	2	3	4	5
33. I think that specialist services' interventions for childhood anxiety disorders are likely to be effective	1	2	3	4	5
a. *If no:* This reduces the likelihood that I will make a referral	1	2	3	4	5
34. I see my role mostly as a sign-poster/referrer for childhood anxiety disorders	1	2	3	4	5
35. Other agencies are responsible for referring childhood anxiety disorders	1	2	3	4	5
a. *If yes:* Name other agencies here					
36. To what extent do the following factors get in the way of your ability to refer children with anxiety disorders to specialist services:	**For the following:**<br>**1 = Not at all**<br>**2= A little**<br>**3 = Somewhat**<br>**4 = Quite a lot**<br>**5 = Very much**				
a. Cultural barriers	1	2	3	4	5
b. Language barriers	1	2	3	4	5
c. Time restrictions	1	2	3	4	5
d. Family reluctance to accept the disorder	1	2	3	4	5
e. Waiting times for specialist services	1	2	3	4	5
f. Limitations in children's communication abilities	1	2	3	4	5
g. Misinformation from parents	1	2	3	4	5
h. My concerns about stigmatising the child	1	2	3	4	5
i. Family concerns about stigma	1	2	3	4	5
j. Lack of training	1	2	3	4	5
k. Lack of experience	1	2	3	4	5
l. Lack of knowledge	1	2	3	4	5
m. Lack of available/accessible treatment	1	2	3	4	5

n. Lack of effective treatment	1	2	3	4	5
Please add any further barriers to referral below if necessary					
o.	1	2	3	4	5
p.	1	2	3	4	5
q.	1	2	3	4	5
Please use this box to add any additional comments you may have					
