# Supplementary material for: Barriers to and facilitators of the identification, management and referral of childhood anxiety disorders in primary care: a survey of general practitioners in England
Source: BMJ Open. 2019 Apr 23;9(4):e023876. doi: 10.1136/bmjopen-2018-023876 (PMC6501977; doi:10.1136/bmjopen-2018-023876)
Supplement: Author's manuscript [file bmjopen-2018-023876.draft_revisions.pdf]

BMJ Open is committed to open peer review. As part of this commitment we make the peer review history of every article we publish publicly available.

When an article is published we post the peer reviewers' comments and the authors' responses online. We also post the versions of the paper that were used during peer review. These are the versions that the peer review comments apply to.

The versions of the paper that follow are the versions that were submitted during the peer review process. They are not the versions of record or the final published versions. They should not be cited or distributed as the published version of this manuscript.

BMJ Open is an open access journal and the full, final, typeset and author-corrected version of record of the manuscript is available on our site with no access controls, subscription charges or pay-per-view fees (http://bmjopen.bmj.com).

If you have any questions on BMJ Open's open peer review process please email info.bmjopen@bmj.com

BMJ Open

# Barriers and facilitators to identification and management of childhood anxiety disorders in primary care: a survey of General Practitioners


| | |
|---|---|
| Journal: | *BMJ Open* |
| Manuscript ID | bmjopen-2018-023876 |
| Article Type: | Research |
| Date Submitted by the Author: | 01-May-2018 |



| | |
|---|---|
| Complete List of Authors: | O'Brien, Doireann; University of Reading, Psychology & Clinical Language Sciences<br>Harvey, Kate; University of Reading, Psychology<br>Creswell, Cathy; University of Reading, Psychology & Clinical Language Sciences |



| | |
|---|---|
| Keywords: | Anxiety disorders < PSYCHIATRY, PRIMARY CARE, Child & adolescent psychiatry < PSYCHIATRY |


SCHOLARONE™
Manuscripts

# Barriers and facilitators to identification and management of childhood anxiety disorders in primary care: a survey of General Practitioners

Doireann O'Brien,[1] Kate Harvey,[1*], Cathy Creswell [1]

[1] School of Psychology and Clinical Language Sciences, University of Reading, UK

*Correspondence to Associate Professor Kate Harvey (k.n.harvey@reading.ac.uk)



For peer review only - http://bmjopen.bmj.com/site/about/guidelines.xhtml

## Abstract

**Objectives:** Although anxiety disorders are the most common emotional disorders in childhood and are associated with a broad range of negative outcomes, only a minority of affected children receive professional support. In the UK, General Practitioners(GPs) are seen as "gate-keepers" to mental health services. The aim of this study was to examine the extent to which GPs experience barriers and facilitators to identifying, managing and accessing specialist services for these disorders, as well as factors associated with GPs' confidence.

**Design & setting**: Cross-sectional, self-report questionnaire in primary care, addressing identification, management and access to specialist services for children(under 12 years) with anxiety disorders

**Participants:** 971 GPs in England

**Primary outcomes:** The primary outcomes for this research was the extent to which GPs felt confident a)identifying and b)managing anxiety disorders in children under 12 years.

**Results:** Only 51% and 13% of GPs felt confident identifying and managing child anxiety disorders respectively. A minority believed that their training in identification(21%) and management(10%) was adequate. Time restrictions inhibited identification and management, and long waiting times was a barrier to accessing specialist services. Being female(Ex(B)=1.4) and being in a less deprived practice(Ex(B)=1.1) were associated with higher confidence identifying anxiety disorders in children. Being a parent of a child over the age of 5(Ex(B)=1.9) and being in a less deprived practice(Ex(B)=1.1) were associated with higher confidence in management. Receipt of psychiatric or paediatric training was not significantly associated with GP confidence.

**Conclusions**: GPs believe that they have a role in identifying and managing child anxiety disorders, however their confidence appears to be related to their personal experience and the context in which they work, rather than their training, highlighting the need to strengthen GP training and facilitate access to resources and services to enable them to support children with these common but debilitating conditions.


**Funding:** This work was supported by grant a National Institute for Health Research 'Research Professorship' to CC (NIHR-RP-2014-04-018). The views expressed are those of the authors and not necessarily those of the NHS, the NIHR or the Department of Health.

**Competing Interests:** None

**Word count:** 3620



For peer review only - http://bmjopen.bmj.com/site/about/guidelines.xhtml

## Strengths and limitations of this study

- A key strength of the study is the inclusion of a large, nationally representative group of GPs.
- In addition to this, we incorporated the findings from a recent systematic review, qualitative study and GP feedback to inform the development of the questionnaire, to ensure that the questions were meaningful to the GPs and that the most pertinent questions were asked.
- Unlike many previous studies, the questionnaire distinguished between different stages of the primary care process (i.e. identification, management, referral to specialist services). This allowed pinpointing of where issues are most problematic.
- These strengths need to be considered in the light of various limitations. Although the sample had similar demographic characteristics to GPs across England and we recruited a substantial proportion of GPs who did not consider themselves ‘research active’, no data were available on those who chose not to participate and we cannot exclude the possibility of a self-selection bias influencing the findings.
- We also did not assess GPs’ abilities to accurately identify anxiety disorders in children and cannot rule out the possibility that in some cases GPs answered questions in relation to mental health disorders other than anxiety.

**Keywords:** Primary Health Care, General Practice, Child Mental Disorders, Access to Health Care, Barriers, Anxiety Disorders

For peer review only

## Introduction

Anxiety disorders are the most common mental health condition(1), with a lifetime prevalence of 28.8%(2) and a median age of onset of 11 years(2). As such, they are the most common emotional disorders in childhood(3, 4) , with worldwide prevalence rates of 6.5% in children and adolescents(5) and are associated with an increased risk of subsequent mental health problems, substance abuse and poor educational attainment(6, 7). This level of impairment results in a high economic and societal burden(8), with anxiety disorders being the leading cause of years lived with disability (YLDs)(9). As such, there is a clear need for early identification and access to evidence-based interventions(10). Effective treatments for childhood anxiety disorders exist(11, 12), however, only a minority of affected children access this support, with lower rates of treatment access than externalizing disorders (such as ADHD or Conduct Disorder) and mood disorders(13, 14).

Two-thirds of British children see their General Practitioner (GP) at least once a year(15) and there has been a steady rise in the number of children and young people presenting in primary care with mental health difficulties(16, 17). Furthermore, GPs are often the first medical professionals that families see when they have mental health concerns and are in a position to develop strong relationships with families, in a non-stigmatising setting(18). As such, they are well placed to support families, and are as often seen as "gate-keepers" to accessing mental health services(19, 20). However, many GPs report a lack of confidence in their competence and skills in child and adolescent mental health, feel that they need further training, particularly in the development of mental health disorders in childhood and adolescence as their current provision is very focused on adults(21), and believe that their role in this area requires further research and definition(22). In addition, they face barriers such as insufficient resources when managing mental health conditions in primary care and extensive waiting lists for specialist services(23). Although GPs are the professional group who make the most referrals to Child and Adolescent Mental Health Services (CAMHS), the likelihood that their referrals will be rejected is very high. The reason for this is unclear, but the corresponding qualitative interviews in this study reflect issues with GP detection of mental health problems in this age group and lack of clarity regarding thresholds for CAMHS amongst GPs, associated with a lack decision making aids for child and adolescent mental health disorders**.**(24; pg 9).

The identification and management of childhood anxiety disorders may present a particular challenge for GPs due to their broad clinical presentation, frequent co-morbidity with other mental health problems(2) and the common reliance on parents/caregivers to seek help(25). Indeed, anxiety disorders are known to be less likely to be recognized by primary health care professionals than other childhood mental health problems(26), potentially due to anxiety disorders being seen as less burdensome for the parents than externalizing disorders (27). In a recent qualitative study, there was wide variation in GPs' confidence managing anxiety disorders in (pre-adolescent) children, with many GPs



For peer review only - http://bmjopen.bmj.com/site/about/guidelines.xhtml

describing feeling ill-equipped to manage and support children with these disorders(28). While many of the barriers they described were similar to those experienced by GPs in relation to other mental health problems in children (e.g. time constraints, difficulties accessing specialist help), GPs highlighted particular difficulties distinguishing anxiety disorders from physical conditions. It was also notable that they attributed their confidence in managing childhood anxiety disorders not only to factors such as training background and practice characteristics but also to whether they were a parent themselves.

Given the substantial burden that anxiety disorders in pose in childhood, and beyond, successful early identification and appropriate management is imperative. However, as previous research has shown, GPs face numerous internal and external barriers in dealing with mental health disorders, with some early indication that anxiety disorders may bring specific challenges. This research aims to build on the findings of the previous qualitative work(28) to ascertain a more representative and generalisable view of these issues throughout the country. In addition to this, the study aims to develop understanding of factors that influence GPs confidence in dealing with these disorders.

*Research questions*

RQ1: i) What are the barriers and facilitators to (a) identification, (b) management and (c) referral to specialist services for pre-adolescent children with anxiety disorders for General Practitioners in England?

RQ2 ii) What practice and personal factors predict GPs' confidence in identifying and managing childhood anxiety disorders?



For peer review only - http://bmjopen.bmj.com/site/about/guidelines.xhtml

## Methods

**Procedure**

Ethical approval for this study was granted by the School of Psychology & Language Science Ethics Board at the University of Reading. Approval was also granted by the Health Research Authority to conduct the study with NHS staff.

Participants completed a 50 item self-report online questionnaire (approx. 15 mins) which contained three sections, addressing a) identification, b) management and c) access to specialist services for children under 12 years with a suspected anxiety disorder (see Appendix 1). Free-text boxes for additional barriers were included at the of each section, and at the end of the survey for any further comment. The content of the survey was informed by findings from a preceding systematic review(23) and qualitative study(28) which highlighted the utility of separating the stages of the primary care process. The questions were developed through an iterative process incorporating feedback from an academic General Practitioner (GP) and the research team.

The first half of each section contained questions about the GP's confidence and beliefs (e.g. "I feel confident identifying anxiety disorders in children under 12 years"). Based on the previous qualitative research, endorsement of these items indicated facilitators (and is labelled as such in the results section). The second half of each section asked GPs to rate external barriers they faced (e.g. "language barriers"). Responses to all items were rated on a 5 point Likert scale (e.g. from 'completely disagree' to 'completely agree', or "not at all" to "very much").

GPs provided information on gender, age, number of years qualified, whether they had completed psychiatric or paediatric training, whether were a parent (including child's age), and whether they considered themselves 'research active'. Information on their practice population, such as size, socio-economic-status and ethnic diversity was obtained via their practice postcode.

**Participants**

*A priori* sample size estimates (https://www.qualtrics.com/blog/calculating-sample-size/) indicated that at least 381 General Practitioners were required to estimate the frequency of particular barriers with 95% confidence intervals, based on a total population of approx. 41,985 GPs in England(29), and to allow for robust analyses using a logistic regression with 10 predictor variables(30).

Participants were GPs currently working in the National Health Service. There were no exclusion criteria. GPs were recruited through the National Institute for Health Research (NIHR) Clinical Research Networks (CRNs). Recruitment methods varied by CRN, from mass emails to



For peer review only - http://bmjopen.bmj.com/site/about/guidelines.xhtml

all registered GPs in the region, to more targeted recruitment, including talks at GP forums. Multiple GPs per practice were allowed. Recruitment took place from the 15th July 2016 – 6th Feb 2017 and covered 12 CRNS (out of a possible 15). CRNs contacted GPs within their regions via an email which contained a link to the survey. Paper copies were available on request.

Participation in this study was anonymous, although GPs' surgery postcode was requested to retrieve demographic information about the practice, and for CRN recruitment purposes. All participants were required to give informed consent.

**Patient and public involvement**

No patients were involved in this research. One academic GP was involved in piloting the questionnaire.

**Statistical Methods**

Statistical analyses were performed using IBM SPSS(V22)(31). As there was little missing data, cases which had missing data were excluded from analysis (number of cases missing per variable ranged between 7-20). To address research question 1, frequencies, including confidence intervals, are reported for items on the questionnaire. To address research question 2, logistic regression analyses were conducted with the dependent variables of confidence a) identifying[2] and b) managing[2] anxiety disorders in children under 12 years (in binary form; 'agree' & 'completely agree' collapsed). The following predictors were included: patient population size[1], deprivation decile of practice (0-10, with 10 being the least deprived)[1], ethnicity of patient population (% non- BAME)[1], psychiatric/ paediatric rotation completed as part of GP training[2], gender[2], number of years since qualification as a GP[2] and whether they were a parent of a child over the age of 5 years[2] (i.e. have personal experience of parenting a school-aged child).

The free-text comments were content-coded and organised into broad categories.

1 Available on Public Health England
[2] Self-report



For peer review only - http://bmjopen.bmj.com/site/about/guidelines.xhtml

## Results

A total of 971 GPs completed the questionnaire. See Table 1 for demographic information for a breakdown of participant numbers by Clinical Research Networks.

Table 1. Characteristics of GPs and their practices.

| Variable | | Missing |
|---|---|---|
| *GP* | | |
| % female | 52.70% | 9 |
| Number of years qualified | M = 14.9 (Range: <1 - 43) | 7 |
| Psychiatric rotation | 47.30% | 16 |
| Paediatric rotation | 73.50% | 11 |
| Child over 5 years old | 72% | 9 |
| Referred a child to specialist services for an anxiety disorder more than 5 times in last 5 years | 58.8 | |
| *Practice* | | |
| Number of registered patients | M = 12,009 (Range: 1503 - 55499) | 19 |
| SES of patients (deprivation decile) | M = 6.7 (Range: 1-10) | 19 |
| % non-white ethnic groups | M = 7% (Range: .8 - 72.2) | 20 |

i) What are the barriers and facilitators to the identification, management and referral of children, under the age of 12 years, with anxiety disorders for General Practitioners in the UK?

Table 2. (below) reports the facilitators and barriers to identification, management and referral, as reported by GPs. A composite of those who agreed and strongly agreed, as well as those who disagreed and strongly disagreed, is presented for each item, in addition to the 95% confidence interval.



For peer review only - http://bmjopen.bmj.com/site/about/guidelines.xhtml

The table demonstrates that although GPs feel that it is their responsibility and feel comfortable discussing anxiety disorders with families, inadequate training, as well as time restrictions and a lack of accessible treatment pose the biggest barriers for GPs in identification of childhood anxiety disorders. A lack of confidence, training, resources and time were the biggest barriers to management. GPs believed that specialist services were effective but waiting times posed the largest barrier to access and less than 10% of GPs felt that they had a relationship with these services. Stigma, language and culture issues were not seen as barriers to GPs' identification, management of referral.

Table 2. GP endorsement of facilitators and barriers to the identification, management and referral of childhood anxiety disorders

| **Item** | **% "agree" + "completely agree"** | **95% confidence interval** | | **% "disagree" + "completely disagree"** | **95% confidence interval** | |
|---|---|---|---|---|---|---|
| ***Identification of anxiety disorders in children under 12 years*** | | *Lower* | *Upper* | | *Lower* | *Upper* |
| *Facilitators* | | | | | | |
| Feel confident in ability | 53 | 50.78 | 57.06 | 12 | 9.91 | 13.99 |
| Believe training was adequate | 21 | 19.09 | 24.29 | 48 | 44.68 | 50.98 |
| Comfortable broaching idea of anxiety disorders with the child | 80 | 77.72 | 82.74 | 7 | 5.14 | 8.30 |
| Comfortable broaching idea of anxiety disorders with the family | 88 | 86.81 | 90.79 | 3 | 1.92 | 4.08 |
| Believe it is part of a GP's responsibility | 93 | 91.60 | 94.78 | 1 | 0.54 | 1.93 |
| *Endorsement of barriers* | **% "quite a lot" + "very much"** | | | **% "a little" + "not at all"** | | |
| Limitations in children's communication abilities | 21 | 19.05 | 24.24 | 33 | 29.93 | 35.84 |
| Misinformation from parents | 27 | 24.06 | 29.65 | 35 | 32.52 | 38.55 |



For peer review only - http://bmjopen.bmj.com/site/about/guidelines.xhtml

| | | | | | | |
|---|---|---|---|---|---|---|
| Concerns about stigmatising the child | 10 | 8.68 | 12.56 | 69 | 66.77 | 72.56 |
| Family concerns about stigma | 16 | 13.60 | 18.21 | 60 | 57.25 | 63.41 |
| Cultural barriers | 17 | 14.46 | 19.17 | 58 | 54.89 | 61.11 |
| Language barriers | 18 | 15.83 | 20.69 | 64 | 61.38 | 67.41 |
| Family reluctance to accept disorder | 20 | 17.73 | 22.80 | 43 | 39.80 | 46.03 |
| Time restrictions | 67 | 63.91 | 69.84 | 14 | 12.33 | 1.677 |
| Lack of available/accessible treatment | 68 | 98.34 | 99.61 | 15 | 12.91 | 17.42 |
| Lack of effective treatment | 45 | 42.53 | 48.80 | 27 | 23.91 | 29.48 |
| ***Management of children under 12 years with anxiety disorders*** | **% “agree” + “completely agree”** | | | **% “disagree” + “completely disagree”** | | |
| *Facilitators* | | | | | | |
| Feel confident in ability | 13 | 11.32 | 15.64 | 51 | 48.71 | 55.02 |
| Believe training was adequate | 10 | 8.29 | 12.09 | 66 | 63.56 | 69.50 |
| Comfortable discussing management strategies with the family | 52 | 49.33 | 55.63 | 20 | 17.32 | 22.34 |
| Comfortable discussing management strategies with the child | 46 | 43.29 | 49.58 | 22 | 19.21 | 24.42 |
| Aware of resources to aid families | 24 | 21.54 | 26.95 | 75 | 73.05 | 78.46 |
| Believe having a relationship with a family is beneficial | 94 | 92.54 | 95.52 | <1 | 0.39 | 1.66 |
| Aware of local agencies to support children and their families | 65 | 63.06 | 69.03 | 17 | 15.09 | 19.89 |



For peer review only - http://bmjopen.bmj.com/site/about/guidelines.xhtml

| | | | | | | |
|---|---|---|---|---|---|---|
| Believe it is part of a GP's role | 69 | 66.70 | 72.49 | 10 | 8.01 | 11.77 |
| *Endorsement of barriers* | **% "quite a lot" + "very much"** | | | **% "a little" + "not at all"** | | |
| Cultural barriers | 14 | 12.01 | 16.43 | 60 | 58.72 | 64.86 |
| Language barriers | 16 | 13.98 | 18.65 | 64 | 61.32 | 67.37 |
| Time restrictions | 72 | 70.15 | 75.76 | 11 | 9.68 | 13.73 |
| Family reluctance to accept the disorder | 19 | 16.78 | 21.76 | 46 | 43.38 | 49.68 |
| Limitations in children's communication abilities | 18 | 15.80 | 20.67 | 43 | 40.60 | 46.86 |
| Misinformation from parents | 18 | 15.55 | 20.40 | 50 | 47/78 | 54.09 |
| Concerns about stigmatising the child | 7 | 5.91 | 9.25 | 71 | 69.31 | 74.98 |
| Family concerns about stigma | 11 | 9.23 | 13.21 | 63 | 60.58 | 66.66 |
| ***Referral of children under 12 years with anxiety disorders*** | **% "agree" + "completely agree"** | | | **% "disagree" + "completely disagree"** | | |
| *Facilitators* | | | | | | |
| Long waiting times reduce the likelihood of making a referral | 50 | 47.37 | 53.66 | 35 | 32.65 | 38.68 |
| Parental pressure increases the likelihood of making a referral | 85 | 83.18 | 87.62 | 5 | 4.02 | 6.87 |
| Have a relationship with local specialist services | 6 | 4.30 | 7.24 | 79 | 76.49 | 81.62 |
| Believe that specialist services' interventions are effective | 78 | 75.63 | 80.83 | 4 | 3.22 | 5.84 |
| *Endorsement of barriers* | **% "quite a lot" + "very much"** | | | **% "a little" + "not at all"** | | |

For peer review only

| | | | | | | |
|---|---|---|---|---|---|---|
| Long waiting times | 89 | 87.03 | 90.97 | 3 | 1.91 | 4.04 |
| Cultural barriers | 8 | 6.22 | 9.65 | 76 | 75.11 | 80.38 |
| Language barriers | 8 | 6.83 | 10.40 | 77 | 76.49 | 81.66 |
| Time restrictions | 30 | 28.26 | 34.14 | 51 | 49.40 | 55.73 |
| Family reluctance to accept the disorder | 12 | 10.47 | 14.68 | 62 | 60.68 | 66.78 |
| Limitations in children's communication abilities | 6 | 4.91 | 8.03 | 70 | 68.19 | 73.93 |
| Misinformation from parents | 9 | 7.83 | 11.59 | 66 | 64.11 | 70.06 |
| Concerns about stigmatising the child | 6 | 4.55 | 7.57 | 78 | 76.94 | 82.06 |
| Family concerns about stigma | 8 | 6.71 | 10.24 | 73 | 71.80 | 77.32 |
| Lack of available/accessible treatment | 54 | 51.78 | 58.10 | 28 | 25.49 | 31.22 |

ii) What factors predict a GPs confidence identifying managing this disorder?

A: The regression model (see Table 3) was a significant fit overall ($\chi^2$ (1) = 16.7, p < .05). Being female was significantly associated with higher confidence identifying anxiety disorders in children, (Ex(B) = 1.4), as was being in a less deprived practice (Ex(B) = 1.1). GP psychiatric and paediatric training, as well as years of experience and parenthood did not have any impact on their confidence, nor did size or ethnicity of their practice population.

Table 3. Regression model of factors which predict a GP's confidence identifying childhood anxiety disorders

| | B | Exp(B) | 95% C.I. for EXP(B) | |
|---|---|---|---|---|
| | | | Lower | Upper |
| *GP* | | | | |
| Gender | .352* | 1.423 | 1.090 | 1.856 |
| Psychiatry training | .147 | 1.158 | .889 | 1.510 |
| Paediatric training | .155 | 1.168 | .861 | 1.5834 |

For peer review only



For peer review only - http://bmjopen.bmj.com/site/about/guidelines.xhtml

| | | | | |
|---|---|---|---|---|
| Years qualified | -.004 | .996 | .980 | 1.012 |
| Child over 5 years | .163 | 1.177 | .841 | 1.656 |
| *Practice* | | | | |
| No. of patients | .000 | 1.000 | 1.000 | 1.000 |
| SES (deprivation decile) | .062* | 1.064 | 1.006 | 1.125 |
| % non-white ethnic groups | .008 | 1.008 | .995 | 1.021 |
| Constant | .081 | .922 | | |

* Note. $R^2$ =. 01 (Hosmer & Lemeshow) .01 (Cox & Snell) .03 (Nagelkerke). Model $\chi2( 1) = 17.4$, $p < .05$.

For peer review only

(ii) What factors predict a GPs confidence managing this disorder?

B: The regression model (see Table 4) was significant overall ($\chi^2(1) = 18.2$, $p < .05$.). Having a child over the age of 5 was significantly associated with greater confidence managing anxiety disorders in children (Ex(B)=1.99), as was being in a less deprived practice (Ex(B)=1.1). GP psychiatric and paediatric training, as well as years of experience and gender did not have any impact on their confidence, nor did size or ethnicity of their practice population.

Table 4. Regression model of factors which predict a GP's confidence managing childhood anxiety disorders

| | B | Exp(B) | 95% C.I. for EXP(B) | |
|---|---|---|---|---|
| | | | Lower | Upper |
| *GP* | | | | |
| Gender | -.239 | .787 | .531 | 1.168 |
| Psychiatry training | .273 | 1.314 | .887 | 1.947 |
| Paediatric training | .218 | 1.244 | .766 | 2.019 |
| Years qualified | .002 | 1.002 | .979 | 1.027 |
| Child over 5 years | .687* | 1.987 | 1.132 | 3.489 |
| *Practice* | | | | |
| No. of patients | .000 | 1.000 | 1.000 | 1.000 |

| | | | | |
|---|---|---|---|---|
| SES (deprivation decile) | .090* | 1.094 | 1.004 | 1.193 |
| % non-white ethnic groups | .015 | 1.015 | .997 | 1.034 |
| Constant | -1.847 | .158 | | |

* Note. $R^2$ =. 05 (Hosmer & Lemeshow) .02 (Cox & Snell) .04 (Nagelkerke). Model $\chi 2( 1) = 20.23$, p = .01.

For peer review only

## Discussion

*Summary*

Although most GPs believe that it is their responsibility to identify and manage anxiety disorders in children, only half of those surveyed felt confident identifying, and only 13% felt confident managing these disorders. These figures may not be surprising given that only 21% of GPs believed their training in identification was adequate, just 10% believed that their training in management of anxiety disorders was adequate, and the vast majority were not aware of any resources to provide families. Time restrictions also posed a barrier for both identification and management. Although 78% of GPs believed that specialist services' interventions were likely to be effective, long waiting times and lack of accessible treatments were overwhelmingly endorsed as a barrier to accessing specialist services and 79% felt that they did not have a relationship with such services. Free-text comments for this section highlighted that GPs felt CAMHS referral criteria were unclear, and often had very high thresholds. However, notably 85% indicated that referral was significantly increased by parental pressure, demonstrating the need for parents to be strong advocates for their children in order to access specialist services. Interestingly, although previous literature reported stigma surrounding mental health as a barrier for GPs, this was not the case for the GPs in this research.

Female GPs and those in less deprived practices were more confident identifying child anxiety disorders. Notably, GPs who had completed psychiatric or paediatric training were not more confident in identification, reinforcing the commonly reported view that the training received was inadequate. In terms of management, GPs in less deprived practices and those who were parents of a child over the age of five were more confident. This latter finding was particularly striking and was consistent with our previous qualitative work that suggested that GPs rely on their own personal experience, as their professional training was largely viewed as inadequate, to manage these disorders(28).

*Strengths and limitations*

One of the key strengths of the study is the inclusion of a large, nationally representative group of GPs. Another is that it used findings from a recent systematic review(23), qualitative study(28) and GP feedback to inform the development of the questionnaire, to ensure that the questions were meaningful to the GPs and that the most pertinent questions were asked. This appeared to have been successful as the free-text responses mainly reinforced the findings from the main questionnaire and little new information was added. Unlike many previous studies, the questionnaire distinguished between different stages of the primary care process (i.e. identification, management, referral to specialist services). This allows pinpointing of where issues are most problematic, for example, time restrictions were a barrier for identification and management, but much less so for referral. We can also see that GPs feel significantly more confident identifying anxiety disorders than managing them.



For peer review only - http://bmjopen.bmj.com/site/about/guidelines.xhtml

These strengths need to be considered in the light of various limitations. Efforts were made to recruit a number of GPs to pilot the survey, however only one responded, which is a limitation of the study. Although the sample had similar demographic characteristics to GPs across England and we recruited a substantial proportion of GPs (n= 443; 46%) who did not consider themselves 'research active', no data were available on those who chose not to participate and we cannot exclude the possibility of a self-selection bias influencing the findings. We also did not assess GPs' abilities to accurately identify anxiety disorders in children and cannot rule out the possibility that in some cases GPs answered questions in relation to mental health disorders other than anxiety. Most free text comments related to questions asked in the survey, however an additional area that GPs raised in the comments was the role of others in identification of anxiety disorders and for referrals to specialist services, in particular, schools and health visitors. A final limitation of this study is the lack of distinction between anxiety disorders, which are not a homogenous group, as well as the fact that we focused on anxiety disorders as a distinct condition, despite the fact that they are often co-morbid with other psychological and physical health disorders.

*Comparison with previous research*

The specific focus on anxiety disorders is a further strength of this research given the specific challenges faced with this condition, and this contrasts with much of the previous work in this area which does not distinguish between mental health disorders. Many of the findings in this study echo previous qualitative findings(24, 28), such as the positive impact of being a parent on confidence managing child anxiety disorders and issues associated with long waiting times and poor access to specialist services. However, although some of the GPs interviewed in the previous research seemed to have some uncertainty regarding their role in the identification and management of this condition, it appeared that the majority of GPs surveyed believed that this is their responsibility, at least in part. This research also reinforced a recent stem4 report which found that GPs had serious concerns about accessing specialist help for young people with mental health disorders(32). The issue of poor access to services and long waiting times are common across the mental health literature, but this study highlights a number of novel findings, such as the widespread lack of confidence in management of childhood anxiety disorders, and the stronger role of personal experience of being a parent than access to relevant specialist training in GP confidence managing childhood anxiety disorders.

*Implications for clinicians, policy-makers and researchers*

The focus of this research was to investigate barriers, from a GP's perspective, however in order to fully understand how to increase access to appropriate support for childhood anxiety disorders, future studies could seek to capture objective data (e.g. referral data), in order to corroborate the questionnaire findings. This would be useful to understand whether self-report correlates with actual behavior. Furthermore, providing GPs



For peer review only - http://bmjopen.bmj.com/site/about/guidelines.xhtml

with vignettes describing children with and without anxiety disorders may be useful to determine whether a GP's perception of their confidence is related to their actual identification abilities.

This research strongly suggests that GPs' current training in childhood anxiety disorders is inadequate, and highlights the need to place increased emphasis on training, both in medical school training and beyond, to boost confidence and develop skills. This training should be meaningful for GPs and firmly grounded in the context of primary care, as well as focused specifically on child and adolescent populations. This supports recommendations from the RCGP that GPs should receive specialist-led training in child health and mental health problems(33). It is encouraging that the majority of GPs surveyed see identifying and managing this condition as part of their role, in addition to other organisations (e.g. schools), but it is possible that a lack of confidence and comfort with the topic matter prevents GPs from fully engaging with a condition(34). Furthermore, as with previous studies in this area, difficulty accessing specialist services in a timely manner poses a significant barrier for GPs who commonly used the free-text comments to express the view that CAMHS' referral thresholds are prohibitively high, and service inclusion criteria are unclear. Increasing GPs confidence in this area, as well as providing them with more effective resources, may encourage and enable GPs to manage many cases in primary care and only refer severe or complex cases, thus alleviating pressure on specialist services.

**Conclusion**

Failing to intervene effectively with childhood anxiety disorders brings negative implications for both individual children and families(6) and increased costs for society(8). GPs consider identification and management of childhood anxiety disorders to be within their role however they face a number of barriers and lack confidence in their abilities. Increased GP training in identification and management of child anxiety disorders may help to strengthen GPs' confidence and ability to effectively support children under the age of 12 with anxiety disorders. Furthermore, the findings suggest that GPs would benefit from increased access to ongoing training and appropriate resources to support identification and early management and to facilitate better communication around access to specialist services for children with anxiety disorders.



For peer review only - http://bmjopen.bmj.com/site/about/guidelines.xhtml

## References

1. Kessler RC, Ruscio AM, Shear K, Wittchen H-U. Epidemiology of anxiety disorders. Behavioral neurobiology of anxiety and its treatment: Springer; 2009. p. 21-35.
2. Kessler R, C.;, Berglund P, Demler O, Jin R, Merikangas KR, Walters EE. Lifetime Prevalence and Age-of-Onset Distributions of DSM-IV Disorders in the National Comorbidity Survey Replication. JAMA Psychiatry. 2005;62(6):593.
3. Lawrence D, Hafekost J, Johnson SE, Saw S, Buckingham WJ, Sawyer MG, et al. Key findings from the second Australian Child and Adolescent Survey of Mental Health and Wellbeing. Aust N Z J Psychiatry. 2016;50(9):876-86.
4. Merikangas KR, He JP, Burstein M, Swanson SA, Avenevoli S, Cui L, et al. Lifetime prevalence of mental disorders in U.S. adolescents: results from the National Comorbidity Survey Replication--Adolescent Supplement (NCS-A). J Am Acad Child Adolesc Psychiatry. 2010;49(10):980-9.
5. Polanczyk GV, Salum GA, Sugaya LS, Caye A, Rohde LA. Annual Research Review: A meta-analysis of the worldwide prevalence of mental disorders in children and adolescents. J Child Psychol Psychiatry. 2015;56(3):345-65.
6. Woodward LJ, Fergusson DM. Life course outcomes of young people with anxiety disorders in adolescence. J Am Acad Child Adolesc Psychiatry. 2001;40(9):1086-93.
7. Knapp M, Snell T, Healey A, Guglani S, Evans-Lacko S, Fernandez JL, et al. How do child and adolescent mental health problems influence public sector costs? Interindividual variations in a nationally representative British sample. J Child Psychol Psychiatry. 2015;56(6):667-76.
8. Fineberg NA, Haddad PM, Carpenter L, Gannon B, Sharpe R, Young AH, et al. The size, burden and cost of disorders of the brain in the UK. J Psychopharmacol. 2013;27(9):761-70.
9. Kassebaum NJ, Arora M, Barber RM, Bhutta ZA, Brown J, Carter A, et al. Global, regional, and national disability-adjusted life-years (DALYs) for 315 diseases and injuries and healthy life expectancy (HALE), 1990-2015: a systematic analysis for the Global Burden of Disease Study 2015. The Lancet. 2016;388(10053):1603.
10. Craske MG, Stein MB. Anxiety. The Lancet. 2016;388(10063):3 048-4059.
11. James AC, James G, Cowdrey FA, Soler A, Choke A. Cognitive behavioural therapy for anxiety disorders in children and adolescents. Cochrane Database Syst Rev. 2013(6):CD004690.
12. Zhen W, H. WSP, Leslie S, Wigdan F, S. MA, Mouaz A, et al. Comparative Effectiveness and Safety of Cognitive Behavioral Therapy and Pharmacotherapy for Childhood Anxiety Disorders A Systematic Review and Meta-analysis. JAMA Paediatrics. 2017.
13. Merikangas KR, He JP, Burstein M, Swendsen J, Avenevoli S, Case B, et al. Service utilization for lifetime mental disorders in U.S. adolescents: results of the National Comorbidity Survey-Adolescent Supplement (NCS-A). J Am Acad Child Adolesc Psychiatry. 2011;50(1):32-45.
14. Essau CA. Frequency and patterns of mental health services utilization among adolescents with anxiety and depressive disorders. Depress Anxiety. 2005;22(3):130-7.



For peer review only - http://bmjopen.bmj.com/site/about/guidelines.xhtml

15. Hippisley-Cox, J, Y. V. Trends in consultation rates in general practice 1995/1996 to 2008/2009: analysis of the QResearch database. . . 2009.
16. Charles J, Harrison CM, Britt H. Management of children's psychological problems in general practice 1970-1971, 1990-1991 and 2008-2009. Australian & New Zealand Journal of Psychiatry. 2011;45(11):976-84.
17. Kelleher KJ, McInerny TK, Gardner WP, Childs GE, Wasserman RC. Increasing identification of psychosocial problems: 1979-1996. Pediatrics. 2000;105(6):1313-21.
18. Roberts JH, Bernard PM. 'Can he have the test for bipolar, doctor? His dad's got it': Exploring the potential of general practitioners to work with children and young people presenting in primary care with common mental health problems - A clinical initiative. Mental Health in Family Medicine. 2012;9(2):115-23.
19. Committee oH. The future of pediatrics: Mental health competencies for pediatric primary care. Pediatrics. 2009;124(1):410-21.
20. Department for Children SaF. *Early intervention: securing good outcomes for all children and young people*Early intervention: securing good outcomes for all children and young people. London: *Department for Children, Schools and Families*Department for Children, Schools and Families; 2010.
21. Roberts JH, Crosland A, Fulton J. "I think this is maybe our Achilles heel..." exploring GPs' responses to young people presenting with emotional distress in general practice: a qualitative study. BMJ Open. 2013;3(9):e002927.
22. Chien A, Coker T, Choi L, Slora E, Bodnar P, Weiley V, et al. What do pediatric primary care providers think are important research questions? A perspective from PROS providers. Ambul Pediatr. 2006;6(6):352-5.
23. O'Brien D, Harvey K, Howse J, Reardon T, Creswell C. Barriers to managing child and adolescent mental health problems: a systematic review of primary care practitioners' perceptions. Br J Gen Pract. 2016:bjgpoct-2016-66-651-harvey-fl-p.
24. Hinrichs S, Owens M, Dunn V, Goodyer I. General practitioner experience and perception of Child and Adolescent Mental Health Services (CAMHS) care pathways: a multimethod research study. BMJ Open. 2012;2(6).
25. Sayal K, Taylor E. Detection of child mental health disorders by general practitioners. British Journal of General Practice. 2004;54(502):348-52.
26. Burke DA, Koot HM, de Wilde A, Begeer S. Influence of Child Factors on Health-Care Professionals' Recognition of Common Childhood Mental-Health Problems. Journal of child and family studies. 2016;25(10):3083-96.
27. Jacobs CO, Loades ME. An investigation into GPs' perceptions of children's mental health problems. Child and Adolescent Mental Health. 2016;21(2):90-5.
28. O'Brien D, Harvey K, Reardon T, Young B, Creswell C. General Practitioners' Experiences of Children with Anxiety Disorders in Primary Care: a Qualitative Study. British Journal of General Practice. 2017.
29. Digital GWTN. General and Personal Medical Services, England September 2015 - March 2016. Health and Social Care Information Centre; 2016.



For peer review only - http://bmjopen.bmj.com/site/about/guidelines.xhtml

30. Peduzzi P, Concato J, Kemper E, Holford TR, Feinstein AR. A simulation study of the number of events per variable in logistic regression analysis. Journal of clinical epidemiology. 1996;49(12):1373-9.

31. Corp I. IBM SPSS Statistics for Windows, Version 22.0. Armonk, NY: IBM Corp; 2013.

32. stem4. “A Time Bomb Waiting to Explode” A Survey of GPs’ Concerns about Mental Health Services for Children and Young People. London; 2016.

33. Practice RCoG. Enhanced and extended GP training progress update. London; 2013.

34. Salmon P, Peters S, Clifford R, Iredale W, Gask L, Rogers A, et al. Why do general practitioners decline training to improve management of medically unexplained symptoms? Journal of General Internal Medicine. 2007;22(5):565-71.

For peer review only

**Author Statement:** All three authors (KH, DOB, CC) were involved in the design of this study. Participant recruitment and analysis was conducted by DOB. The main draft of the manuscript was written by DOB with input by KH and CC.

**Data sharing statement:** Full dataset and statistical codes are available from the corresponding author. 'Data is currently being uploading to the University of Reading research data archive and a link will be provided prior to publication'

For peer review only



For peer review only - http://bmjopen.bmj.com/site/about/guidelines.xhtml

# CADPC

## Consent page

### Children with Anxiety Disorders in Primary Care: the views of General Practitioners II

We invite you to take part in a National Institute of Health Research funded study which focuses on management of childhood anxiety disorders (children under 12 years) by General Practitioners in England. This study explores GPs' experiences of identification, management and referral of these disorders.

**Please note, while some of these questions may seem repetitive, consider whether they are being asked in relation to either identification, management or referral.** Your responses will help inform practice guidelines for children with anxiety disorders.

**Taking part in this study will involve completing a survey, which should take 10-15 minutes to complete. This is open to as many GPs per surgery as desired.**

As a reimbursement for taking part in the study you (or a nominated member of your practice) will have the opportunity to take part in a Webinar about identification and management of childhood anxiety disorders. This module may be used to contribute towards Continued Professional Development points and a certificate of attendance outlining your involvement in important mental health research will be provided. You will also be entered into a prize draw to win one of two £100 Amazon vouchers.

We are happy to answer queries about the study, please contact the researchers for further information:

Doireann O'Brien, B.A., M.Sc.
Email: doireann.obrien@pgr.reading.ac.uk

Professor Cathy Creswell
Tel: +44 (0)118 378 6798

Further details of the study are available as an information sheet. Please find information sheet enclosed.

**All items of the consent form, below, must be initialled to indicate consent.**

For peer review only

I can confirm that I have read and understand the information sheet, have had the opportunity to ask questions and, if applicable, have had these questions answered satisfactorily ⓘ *Required* ☐

I understand that my participation is voluntary, that I can withdraw my consent at any time, without giving a reason and without my professional role being compromised ⓘ *Required* ☐

I understand that the answers to my questions will be stored in a secure location, but will bear no identifying information. I also understand that the data will be kept for 12 months and after this time they will be destroyed ⓘ *Required* ☐

I understand that, should I lose the capacity to consent to be in the study, the research centre will retain any information collected prior to this point ⓘ *Required* ☐

I understand that relevant sections of my data collected during the study, may be looked at by individuals from the University of Reading, from regulatory authorities or from the NHS Trust, where it is relevant to my taking part in this research. I give permission for these individuals to have access to my records ⓘ *Required* ☐

I agree to take part in the study ⓘ *Required* ☐

For peer review only

# SURVEY

| | |
|---|---|
| Age: *(please circle)* 23-35 35-45 45-55 55-65 65+ | |
| Identify my gender as: *(please circle)* MALE FEMALE OTHER | |
| Psychiatric rotation completed as part of GP training: *(please circle)* YES NO | |
| Paediatric rotation completed as part of GP training: *(please circle)* YES NO | |
| Year qualified as a GP: | |
| Practice Name: | |
| Practice Postcode: | |
| Years employed in current Trust: | |
| Would you consider yourself research active? *(please circle)* YES NO | |
| Are you a parent or guardian? *(please circle)* YES NO | |
| If so, what ages are your children: | |
| Compared to other GPs in your practice, do you see more or fewer child patients? *(please circle)* YES NO SIMILAR AMOUNT | |

For peer review only

PLEASE NOTE: **ALL QUESTIONS ARE IN RELATION TO ANXIETY IN CHILDREN AGED 12 & UNDER (i.e. primary school age and below)**

For each item identified below, circle the number to the right that best fits your judgment of its quality.
Use the rating scale to select the number.

| Survey Item | Scale* 1 = completely disagree 2 = disagree 3 = neutral 5 = agree 5 = completely agree *unless otherwise specified | | | | |
|---|---|---|---|---|---|
| **Identification** *These questions are in relation to initially identifying the presence of an Anxiety Disorder in a child* | | | | | |
| 1. How common are anxiety disorders in children in your experience? | 1 in 3 | 1 in 10 | 1 in 15 | 1 in 20 | 1 in 40 |
| 2. How often do you see anxiety disorders in your practice? | | | | | |
| 3. I believe that anxiety disorders are prevalent in children under 12 | 1 | 2 | 3 | 4 | 5 |
| 4. I am confident in my ability to recognise anxiety disorders in children | 1 | 2 | 3 | 4 | 5 |
| 5. I am regularly looking out for anxiety disorders in children | 1 | 2 | 3 | 4 | 5 |
| 6. My training adequately equipped me with the skills to identify anxiety disorders in children | 1 | 2 | 3 | 4 | 5 |
| 7. I use screening tools to aid identification of anxiety disorders in children (Screening tools refer to validated measures used to aid identification of a suspected anxiety disorder) | Yes | | | No | |
| a. *If yes:* I find screening tools useful for the identification of anxiety disorders in children | 1 | 2 | 3 | 4 | 5 |
| b. *If no:* I would find a brief screening tool useful for identification of anxiety disorders in children | 1 | 2 | 3 | 4 | 5 |
| 8. I am comfortable broaching the subject of a potential anxiety disorder with: | | | | | |
| a. the child | 1 | 2 | 3 | 4 | 5 |
| b. the family | 1 | 2 | 3 | 4 | 5 |
| 9. I believe that it is part of my responsibility in my role as a GP to identify anxiety disorders in children | 1 | 2 | 3 | 4 | 5 |
| 10. Other agencies are responsible for identifying childhood anxiety disorders | 1 | 2 | 3 | 4 | 5 |
| a. *If yes*: Name agencies here | | | | | |

For peer review only - http://bmjopen.bmj.com/site/about/guidelines.xhtml

| 11. To what extent do the following factors get in the way of your ability to accurately identify anxiety disorders in children: | **For the following:**<br>**1 = Not at all**<br>**2= A little**<br>**3 = Somewhat**<br>**4 = Quite a lot**<br>**5 = Very much** | | | | |
|---|---|---|---|---|---|
| a. Limitations in children's communication abilities | 1 | 2 | 3 | 4 | 5 |
| b. Misinformation from parents | 1 | 2 | 3 | 4 | 5 |
| c. My concerns about stigmatising the child | 1 | 2 | 3 | 4 | 5 |
| d. Family concerns about stigma | 1 | 2 | 3 | 4 | 5 |
| e. Cultural barriers | 1 | 2 | 3 | 4 | 5 |
| f. Language barriers | 1 | 2 | 3 | 4 | 5 |
| g. Time restrictions | 1 | 2 | 3 | 4 | 5 |
| h. Family reluctance to accept disorder | 1 | 2 | 3 | 4 | 5 |
| i. Lack of training | 1 | 2 | 3 | 4 | 5 |
| j. Lack of experience | 1 | 2 | 3 | 4 | 5 |
| k. Lack of knowledge | 1 | 2 | 3 | 4 | 5 |
| l. Lack of available/accessible treatment | 1 | 2 | 3 | 4 | 5 |
| m. Lack of effective treatment | 1 | 2 | 3 | 4 | 5 |
| Please add any further barriers to identification here if necessary | | | | | |
| n. | 1 | 2 | 3 | 4 | 5 |
| o. | 1 | 2 | 3 | 4 | 5 |
| p. | 1 | 2 | 3 | 4 | 5 |
| q. | | | | | |
| r. | | | | | |

| **Survey Item** | **Scale***<br>**1 = completely disagree**<br>**2 = disagree**<br>**3 = neutral**<br>**5 = agree**<br>**5 = completely agree**<br><br>*unless otherwise specified |
|---|---|

**Management:**

*These questions are in relation to your ongoing management of a childhood Anxiety Disorders, either prior to making a referral or in the absence of a referral*

For peer review only

| | | | | | |
|---|---|---|---|---|---|
| 12. I believe that I have had adequate training to manage anxiety disorders in children | 1 | 2 | 3 | 4 | 5 |
| 13. I have adequate skills to manage anxiety disorders in children | 1 | 2 | 3 | 4 | 5 |
| 14. I feel confident managing anxiety disorders in children | 1 | 2 | 3 | 4 | 5 |
| 15. I am comfortable discussing management strategies for anxiety disorders with: | | | | | |
| a. the child | 1 | 2 | 3 | 4 | 5 |
| b. the family | 1 | 2 | 3 | 4 | 5 |
| 16. I provide families with management tips & strategies for managing anxiety disorders in children | 1 | 2 | 3 | 4 | 5 |
| 17. I am aware of resources such as books/websites/apps to aid families in the management of anxiety disorders in children | Yes | | | No | |
| a. *If yes:* I use resources such as books/websites/apps in the management of childhood anxiety disorders | 1 | 2 | 3 | 4 | 5 |
| b. *If yes:* Please state which ones | | | | | |
| c. *If no:* I would use resources such as books/websites/apps in the management of childhood anxiety disorders | 1 | 2 | 3 | 4 | 5 |
| 18. Are you aware of any online/apps resources for parents concerned about their child's anxiety? | YES | | | NO | |
| a. *If Yes*: please state which ones | | | | | |
| b. *If Yes:* I have sign-posted parents concerned about their child's anxiety to these | 1 | 2 | 3 | 4 | 5 |
| c. *If No:* If I were aware of online/app resources, I would signpost parents to these if they were concerned about their child's anxiety | 1 | 2 | 3 | 4 | 5 |
| 19. It would be important for an online/app resource for childhood anxiety to have an evidence-base in order for me to recommend it | 1 | 2 | 3 | 4 | 5 |
| 20. It would be important to have NHS accreditation of the online/resources for childhood anxiety be in order for me to recommend it | 1 | 2 | 3 | 4 | 5 |
| 21. Having a relationship with a family aids me in the management of anxiety disorders | 1 | 2 | 3 | 4 | 5 |
| 22. I involve the child's school in the management of anxiety disorders | 1 | 2 | 3 | 4 | 5 |
| a. *If yes:* I find this helpful | 1 | 2 | 3 | 4 | 5 |
| 23. I am aware of local agencies available to support children with anxiety disorders and their families | 1 | 2 | 3 | 4 | 5 |
| 24. I believe it is part of my role as a GP to manage anxiety disorders in children | 1 | 2 | 3 | 4 | 5 |
| 25. Other agencies are responsible for managing childhood anxiety disorders | 1 | 2 | 3 | 4 | 5 |
| a. Add other agencies here if applicable | | | | | |

For peer review only

| | For the following:<br>1 = Not at all<br>2= A little<br>3 = Somewhat<br>4 = Quite a lot<br>5 = Very much | | | | |
|---|---|---|---|---|---|
| 26. To what extent do the following factors get in the way of your ability to effectively manage anxiety disorders in children: | | | | | |
| a. Cultural barriers | 1 | 2 | 3 | 4 | 5 |
| b. Language barriers | 1 | 2 | 3 | 4 | 5 |
| c. Time restrictions | 1 | 2 | 3 | 4 | 5 |
| d. Family reluctance to accept the disorder | 1 | 2 | 3 | 4 | 5 |
| e. Limitations in children's communication abilities | 1 | 2 | 3 | 4 | 5 |
| f. Misinformation from parents | 1 | 2 | 3 | 4 | 5 |
| g. My concerns about stigmatising the child | 1 | 2 | 3 | 4 | 5 |
| h. Family concerns about stigma | 1 | 2 | 3 | 4 | 5 |
| i. Lack of training | 1 | 2 | 3 | 4 | 5 |
| j. Lack of experience | 1 | 2 | 3 | 4 | 5 |
| k. Lack of knowledge | 1 | 2 | 3 | 4 | 5 |
| Please add any further barriers to management below if necessary | | | | | |
| l. | 1 | 2 | 3 | 4 | 5 |
| m. | 1 | 2 | 3 | 4 | 5 |
| n. | 1 | 2 | 3 | 4 | 5 |

| Survey Item | Scale*<br>1 = completely disagree<br>2 = disagree<br>3 = neutral<br>5 = agree<br>5 = completely agree<br><br>*unless otherwise specified | | | | |
|---|---|---|---|---|---|
| **Referral**<br>*These questions are in relation to a referral of a childhood Anxiety Disorder to a specialist service (such as Child & Adolescent Mental Health Services)* | | | | | |
| 27. How often in the last 5 years have you referred a child to specialist services for an anxiety disorder? | 0 times | 0-5 times | 5-10 times | 10-15 times | 15+ times |
| 28. Parental pressure increases the likelihood that I would refer a child with an anxiety disorder to specialist services | 1 | 2 | 3 | 4 | 5 |
| 29. anxiety disorders in children will generally resolve itself without treatment | 1 | 2 | 3 | 4 | 5 |
| 30. I seek specialist advice prior to making a referral of a childhood anxiety disorder | 1 | 2 | 3 | 4 | 5 |

For peer review

| | | | | | |
|---|---|---|---|---|---|
| a. *If yes:* I find this helpful | 1 | 2 | 3 | 4 | 5 |
| b. *If no:* I think that I would find this helpful | 1 | 2 | 3 | 4 | 5 |
| 31. I experience long waiting times for referral for a childhood anxiety disorders | 1 | 2 | 3 | 4 | 5 |
| a. *If yes:* Long waiting times reduce the likelihood that I would make a referral to specialist services for childhood anxiety disorders | 1 | 2 | 3 | 4 | 5 |
| 32. I feel like I have a relationship with specialist services for childhood anxiety disorders | 1 | 2 | 3 | 4 | 5 |
| a. *If yes:* This is helpful when making a referral | 1 | 2 | 3 | 4 | 5 |
| b. *If no:* I think this would this be helpful when making a referral | 1 | 2 | 3 | 4 | 5 |
| 33. I think that specialist services' interventions for childhood anxiety disorders are likely to be effective | 1 | 2 | 3 | 4 | 5 |
| a. *If no:* This reduces the likelihood that I will make a referral | 1 | 2 | 3 | 4 | 5 |
| 34. I see my role mostly as a sign-poster/referrer for childhood anxiety disorders | 1 | 2 | 3 | 4 | 5 |
| 35. Other agencies are responsible for referring childhood anxiety disorders | 1 | 2 | 3 | 4 | 5 |
| a. *If yes:* Name other agencies here | | | | | |
| 36. To what extent do the following factors get in the way of your ability to refer children with anxiety disorders to specialist services: | **For the following:**<br>**1 = Not at all**<br>**2= A little**<br>**3 = Somewhat**<br>**4 = Quite a lot**<br>**5 = Very much** | | | | |
| a. Cultural barriers | 1 | 2 | 3 | 4 | 5 |
| b. Language barriers | 1 | 2 | 3 | 4 | 5 |
| c. Time restrictions | 1 | 2 | 3 | 4 | 5 |
| d. Family reluctance to accept the disorder | 1 | 2 | 3 | 4 | 5 |
| e. Waiting times for specialist services | 1 | 2 | 3 | 4 | 5 |
| f. Limitations in children's communication abilities | 1 | 2 | 3 | 4 | 5 |
| g. Misinformation from parents | 1 | 2 | 3 | 4 | 5 |
| h. My concerns about stigmatising the child | 1 | 2 | 3 | 4 | 5 |
| i. Family concerns about stigma | 1 | 2 | 3 | 4 | 5 |
| j. Lack of training | 1 | 2 | 3 | 4 | 5 |
| k. Lack of experience | 1 | 2 | 3 | 4 | 5 |
| l. Lack of knowledge | 1 | 2 | 3 | 4 | 5 |
| m. Lack of available/accessible treatment | 1 | 2 | 3 | 4 | 5 |

For peer review only

| | | | | | |
|---|---|---|---|---|---|
| n. Lack of effective treatment | 1 | 2 | 3 | 4 | 5 |
| Please add any further barriers to referral below if necessary | | | | | |
| o. | 1 | 2 | 3 | 4 | 5 |
| p. | 1 | 2 | 3 | 4 | 5 |
| q. | 1 | 2 | 3 | 4 | 5 |
| Please use this box to add any additional comments you may have | | | | | |

For peer review only

**STROBE 2007 (v4) Statement—Checklist of items that should be included in reports of *cross-sectional studies***

| Section/Topic | Item # | Recommendation | Reported on page # |
|---|---|---|---|
| **Title and abstract** | 1 | (*a*) Indicate the study's design with a commonly used term in the title or the abstract | 1 |
| | | (*b*) Provide in the abstract an informative and balanced summary of what was done and what was found | 2 |
| **Introduction** | | | |
| Background/rationale | 2 | Explain the scientific background and rationale for the investigation being reported | 4-5 |
| Objectives | 3 | State specific objectives, including any prespecified hypotheses | 4-5 |
| **Methods** | | | |
| Study design | 4 | Present key elements of study design early in the paper | 6-7 |
| Setting | 5 | Describe the setting, locations, and relevant dates, including periods of recruitment, exposure, follow-up, and data collection | 6-7 |
| Participants | 6 | (*a*) Give the eligibility criteria, and the sources and methods of selection of participants | 6-7 |
| Variables | 7 | Clearly define all outcomes, exposures, predictors, potential confounders, and effect modifiers. Give diagnostic criteria, if applicable | 6-7 |
| Data sources/ measurement | 8* | For each variable of interest, give sources of data and details of methods of assessment (measurement). Describe comparability of assessment methods if there is more than one group | 6-7 |
| Bias | 9 | Describe any efforts to address potential sources of bias | |
| Study size | 10 | Explain how the study size was arrived at | 6 |
| Quantitative variables | 11 | Explain how quantitative variables were handled in the analyses. If applicable, describe which groupings were chosen and why | 6-7 |
| Statistical methods | 12 | (*a*) Describe all statistical methods, including those used to control for confounding | 6-7 |
| | | (*b*) Describe any methods used to examine subgroups and interactions | 6-7 |
| | | (*c*) Explain how missing data were addressed | 6-7 |
| | | (*d*) If applicable, describe analytical methods taking account of sampling strategy | 6-7 |
| | | (*e*) Describe any sensitivity analyses | |
| **Results** | | | |

For peer review only

| | | | |
|---|---|---|---|
| Participants | 13* | (a) Report numbers of individuals at each stage of study—eg numbers potentially eligible, examined for eligibility, confirmed eligible, included in the study, completing follow-up, and analysed | 8 |
| | | (b) Give reasons for non-participation at each stage | N/a |
| | | (c) Consider use of a flow diagram | N/a |
| Descriptive data | 14* | (a) Give characteristics of study participants (eg demographic, clinical, social) and information on exposures and potential confounders | 8 |
| | | (b) Indicate number of participants with missing data for each variable of interest | 8 |
| Outcome data | 15* | Report numbers of outcome events or summary measures | 8 |
| Main results | 16 | (*a*) Give unadjusted estimates and, if applicable, confounder-adjusted estimates and their precision (eg, 95% confidence interval). Make clear which confounders were adjusted for and why they were included | 8 |
| | | (*b*) Report category boundaries when continuous variables were categorized | N/A |
| | | (*c*) If relevant, consider translating estimates of relative risk into absolute risk for a meaningful time period | N/A |
| Other analyses | 17 | Report other analyses done—eg analyses of subgroups and interactions, and sensitivity analyses | 8 |
| **Discussion** | | | |
| Key results | 18 | Summarise key results with reference to study objectives | 9 |
| Limitations | 19 | Discuss limitations of the study, taking into account sources of potential bias or imprecision. Discuss both direction and magnitude of any potential bias | 9 |
| Interpretation | 20 | Give a cautious overall interpretation of results considering objectives, limitations, multiplicity of analyses, results from similar studies, and other relevant evidence | 10 |
| Generalisability | 21 | Discuss the generalisability (external validity) of the study results | 10-11 |
| **Other information** | | | |
| Funding | 22 | Give the source of funding and the role of the funders for the present study and, if applicable, for the original study on which the present article is based | 2 |

*Give information separately for cases and controls in case-control studies and, if applicable, for exposed and unexposed groups in cohort and cross-sectional studies.

**Note:** An Explanation and Elaboration article discusses each checklist item and gives methodological background and published examples of transparent reporting. The STROBE checklist is best used in conjunction with this article (freely available on the Web sites of PLoS Medicine at http://www.plosmedicine.org/, Annals of Internal Medicine at http://www.annals.org/, and Epidemiology at http://www.epidem.com/). Information on the STROBE Initiative is available at www.strobe-statement.org.

For peer review only

BMJ Open

# Barriers and facilitators to identification and management of childhood anxiety disorders in primary care: a survey of General Practitioners

| | |
|---|---|
| Journal: | *BMJ Open* |
| Manuscript ID | bmjopen-2018-023876.R1 |
| Article Type: | Research |
| Date Submitted by the Author: | 25-Oct-2018 |
| Complete List of Authors: | O'Brien, Doireann; University of Reading, Psychology & Clinical Language Sciences<br>Harvey, Kate; University of Reading, Psychology<br>Creswell, Cathy; University of Reading, Psychology & Clinical Language Sciences |
| <b>Primary Subject Heading</b>: | Mental health |
| Secondary Subject Heading: | Paediatrics, General practice / Family practice |
| Keywords: | Anxiety disorders < PSYCHIATRY, PRIMARY CARE, Child & adolescent psychiatry < PSYCHIATRY |
| | |

SCHOLARONE™
Manuscripts

# Barriers and facilitators to identification and management of childhood anxiety disorders in primary care: a survey of General Practitioners

Doireann O'Brien,[1] Kate Harvey,[1*], Cathy Creswell [1]

[1] School of Psychology and Clinical Language Sciences, University of Reading, UK

*Correspondence to Associate Professor Kate Harvey (k.n.harvey@reading.ac.uk)

For peer review only

## Abstract

**Objectives:** Although anxiety disorders are the most common emotional disorders in childhood and are associated with a broad range of negative outcomes, only a minority of affected children receive professional support. In the UK, General Practitioners(GPs) are seen as "gate-keepers" to mental health services. The aim of this study was to examine the extent to which GPs experience barriers and facilitators to identifying, managing and accessing specialist services for these disorders, as well as factors associated with GPs' confidence.

**Design & setting**: Cross-sectional, self-report questionnaire in primary care, addressing identification, management and access to specialist services for children(under 12 years) with anxiety disorders

**Participants:** 971 GPs in England

**Primary outcomes:** The primary outcomes for this research was the extent to which GPs felt confident a)identifying and b)managing anxiety disorders in children under 12 years.

**Results:** Only 51% and 13% of GPs felt confident identifying and managing child anxiety disorders respectively. A minority believed that their training in identification(21%) and management(10%) was adequate. Time restrictions inhibited identification and management, and long waiting times was a barrier to accessing specialist services. Being female(Ex(B)=1.4) and being in a less deprived practice(Ex(B)=1.1) was associated with higher confidence identifying anxiety disorders in children. Being a parent of a child over the age of 5(Ex(B)=1.9) and being in a less deprived practice(Ex(B)=1.1) was associated with higher confidence in management. Receipt of psychiatric or paediatric training was not significantly associated with GP confidence.

**Conclusions**: GPs believe that they have a role in identifying and managing child anxiety disorders, however their confidence appears to be related to their personal experience and the context in which they work, rather than their training, highlighting the need to strengthen GP training and facilitate access to resources and services to enable them to support children with these common but debilitating conditions.


**Funding:** This work was supported by grant a National Institute for Health Research 'Research Professorship' to CC (NIHR-RP-2014-04-018). The views expressed are those of the authors and not necessarily those of the NHS, the NIHR or the Department of Health.

**Competing Interests:** None
**Word count:** 3620


For peer review only - http://bmjopen.bmj.com/site/about/guidelines.xhtml

## Strengths and limitations of this study

- A key strength of the study is the inclusion of a large, nationally representative group of GPs.
- In addition to this, we incorporated the findings from a recent systematic review, qualitative study and GP feedback to inform the development of the questionnaire, to ensure that the questions were meaningful to the GPs and that the most pertinent questions were asked.
- Unlike many previous studies, the questionnaire distinguished between different stages of the primary care process (i.e. identification, management, referral to specialist services). This allowed pinpointing of where issues are most problematic.
- These strengths need to be considered in the light of various limitations. Although the sample had similar demographic characteristics to GPs across England and we recruited a substantial proportion of GPs who did not consider themselves ‘research active’, no data were available on those who chose not to participate and we cannot exclude the possibility of a self-selection bias influencing the findings.
- We also did not assess GPs’ abilities to accurately identify anxiety disorders in children and cannot rule out the possibility that in some cases GPs answered questions in relation to mental health disorders other than anxiety.

**Keywords:** Primary Health Care, General Practice, Child Mental Disorders, Access to Health Care, Barriers, Anxiety Disorders

For peer review only

## Introduction

Anxiety disorders are the most common mental health condition(1), with a lifetime prevalence of 28.8%(2) and a median age of onset of 11 years(2). As such, they are the most common emotional disorders in childhood(3, 4) , with worldwide prevalence rates of 6.5% in children and adolescents(5) and are associated with an increased risk of subsequent mental health problems, substance abuse and poor educational attainment(6, 7). This level of impairment results in a high economic and societal burden(8), with anxiety disorders being the leading cause of years lived with disability (YLDs)(9). As such, there is a clear need for early identification and access to evidence-based interventions(10). Effective treatments for childhood anxiety disorders exist(11, 12), however, only a minority of affected children access this support, with lower rates of treatment access than externalizing disorders (such as ADHD or Conduct Disorder) and mood disorders(13, 14).

In the UK, two-thirds of children see their General Practitioner (GP) at least once a year(15) and there has been a steady rise in the number of children and young people presenting in primary care with mental health difficulties(16, 17). GPs are often the first medical professionals that families see when they have mental health concerns and are in a position to develop strong relationships with families, in a non-stigmatising setting(18) and are as often seen as "gate-keepers" to accessing mental health services(19, 20). However, many GPs report a lack of confidence in their competence and skills in child and adolescent mental health, feel that they need further training, (21), and believe that their role in this area requires further research and definition(22). In addition, they face barriers such as insufficient resources when managing mental health conditions in primary care and extensive waiting lists for specialist services(23) and a high rate of rejected referrals. The reason for this is unclear, possibly reflecting issues with GP detection of mental health problems in this age group and lack of clarity regarding thresholds for CAMHS amongst GPs, associated with a lack decision making aids for child and adolescent mental health disorders**.**(24).

The identification and management of childhood anxiety disorders may present a particular challenge for GPs due to their broad clinical presentation, frequent co-morbidity with other mental health problems(2) and the common reliance on parents/caregivers to seek help(25). Indeed, anxiety disorders are known to be less likely to be recognized by primary health care professionals than other childhood mental health problems(26), potentially due to anxiety disorders being seen as less burdensome for the parents than externalizing disorders (27). In a recent qualitative study, there was wide variation in GPs' confidence managing anxiety disorders in (pre-adolescent) children, with many GPs describing feeling ill-equipped to manage and support children with these disorders(28). While many of the barriers they described were similar to those experienced by GPs in relation to other mental health problems in children (e.g. time constraints, difficulties accessing specialist help), GPs highlighted particular difficulties



For peer review only - http://bmjopen.bmj.com/site/about/guidelines.xhtml

distinguishing anxiety disorders from physical conditions. It was also notable that they attributed their confidence in managing childhood anxiety disorders not only to factors such as training background and practice characteristics but also to whether they were a parent themselves.

Given the substantial burden that anxiety disorders in pose in childhood, and beyond, successful early identification and appropriate management is imperative. However, as previous research has shown, GPs face numerous internal and external barriers in dealing with mental health disorders, with some early indication that anxiety disorders may bring specific challenges. This research aims to build on the findings of the previous qualitative work(28) to ascertain a more representative and generalisable view of these issues throughout the country. In addition to this, the study aims to develop understanding of factors that influence GPs confidence in dealing with these disorders.

*Research questions*

RQ1: i) What are the barriers and facilitators to (a) identification, (b) management and (c) referral to specialist services for pre-adolescent children with anxiety disorders for General Practitioners in England?

RQ2 ii) What practice and personal factors are associated with GPs' confidence in identifying and managing childhood anxiety disorders?

For peer review only



For peer review only - http://bmjopen.bmj.com/site/about/guidelines.xhtml

## Methods

### Procedure

Ethical approval for this study was granted by the School of Psychology & Language Science Ethics Board at the University of Reading. Approval was also granted by the Health Research Authority to conduct the study with NHS staff.

Participants completed a 50 item self-report online questionnaire (approx. 15 mins) which contained three sections, addressing a) identification, b) management and c) access to specialist services for children under 12 years with a suspected anxiety disorder (see Appendix 1). Free-text boxes for additional barriers were included at the of each section, and at the end of the survey for any further comment. The content of the survey was informed by findings from a preceding systematic review(23) and qualitative study(28) which highlighted the utility of separating the stages of the primary care process. The questions were developed through an iterative process incorporating feedback from an academic General Practitioner (GP) and the research team.

The first half of each section contained questions about the GP's confidence and beliefs (e.g. "I feel confident identifying anxiety disorders in children under 12 years"). Based on the previous qualitative research, endorsement of these items indicated facilitators (and is labelled as such in the results section). The second half of each section asked GPs to rate external barriers they faced (e.g. "language barriers"). Responses to all items were rated on a 5 point Likert scale (e.g. from 'completely disagree' to 'completely agree', or "not at all" to "very much").

GPs provided information on gender, age, number of years qualified, whether they had completed psychiatric or paediatric training, whether were a parent (including child's age), and whether they considered themselves 'research active'. Information on their practice population, such as size, socio-economic-status and ethnic diversity was obtained via their practice postcode.

### Participants

*A priori* sample size estimates (https://www.qualtrics.com/blog/calculating-sample-size/) indicated that at least 381 General Practitioners were required to estimate the frequency of particular barriers with 95% confidence intervals, based on a total population of approx. 41,985 GPs in England(29), and to allow for robust analyses using a logistic regression with 10 predictor variables(30).

Participants were GPs currently working in the National Health Service. There were no exclusion criteria. GPs were recruited through the National Institute for Health Research (NIHR) Clinical Research Networks (CRNs). Recruitment methods varied by CRN, from mass emails to all registered GPs in the region, to more targeted recruitment, including talks at GP forums. Multiple GPs per practice were allowed. Recruitment took place from the 15th July 2016 – 6th


For peer review only - http://bmjopen.bmj.com/site/about/guidelines.xhtml

Feb 2017 and covered 11 CRNS (out of a possible 15). CRNs contacted GPs within their regions via an email which contained a link to the survey. Paper copies were available on request.

Participation in this study was anonymous, although GPs' surgery postcode was requested to retrieve demographic information about the practice, and for CRN recruitment purposes. All participants were required to give informed consent.

**Patient and public involvement**

No patients were involved in this research. One academic GP was involved in piloting the questionnaire.

**Statistical Methods**

Statistical analyses were performed using IBM SPSS(V22)(31). As there was little missing data, cases which had missing data were excluded from analysis (number of cases missing per variable ranged between 7-20). To address research question 1, frequencies, including confidence intervals, are reported for items on the questionnaire. To address research question 2, logistic regression analyses were conducted with the dependent variables of confidence a) identifying[2] and b) managing[2] anxiety disorders in children under 12 years. These dependent variables were derived from the questions: 'I am confident identifying anxiety disorders' and 'I am confident managing anxiety disorders'. These questions were transformed into a binary format by collapsing; 'agree' and 'completely agree' together and 'neutral', 'disagree' and 'completely disagree' together. The following predictors were included: patient population size[1], deprivation decile of practice (0-10, with 10 being the least deprived)[1], ethnicity of patient population (% non- BAME)[1], psychiatric/ paediatric rotation completed as part of GP training[2], gender[2], number of years since qualification as a GP[2] and whether they were a parent of a child over the age of 5 years[2] (i.e. have personal experience of parenting a school-aged child). These factors were chosen based on previous qualitative research which indicated the potential influence they have on a GP's confidence.

The free-text comments were content-coded and organised into broad categories.

1 Available on Public Health England

[2] Self-report



For peer review only - http://bmjopen.bmj.com/site/about/guidelines.xhtml

## Results

A total of 971 GPs completed the questionnaire. See Table 1 for demographic information for a breakdown of participant numbers by Clinical Research Networks.

Table 1. Characteristics of GPs and their practices.

| Variable | | Missing |
|---|---|---|
| *GP* | | |
| % female | 52.70% | 9 |
| Number of years qualified | M = 14.9 (Range: <1 - 43) | 7 |
| Psychiatric rotation | 47.30% | 16 |
| Paediatric rotation | 73.50% | 11 |
| Child over 5 years old | 72% | 9 |
| Referred a child to specialist services for an anxiety disorder more than 5 times in last 5 years | 58.8 | |
| *Practice* | | |
| Number of registered patients | M = 12,009 (Range: 1503 - 55499) | 19 |
| SES of patients (deprivation decile) | M = 6.7 (Range: 1-10) | 19 |
| % non-white ethnic groups | M = 7% (Range: .8 - 72.2) | 20 |

i) What are the barriers and facilitators to the identification, management and referral of children, under the age of 12 years, with anxiety disorders for General Practitioners in the UK?

Table 2. (below) reports the facilitators and barriers to identification, management and referral, as reported by GPs. A composite of those who agreed and strongly agreed, as well as those who disagreed and strongly disagreed, is presented for each item, in addition to the 95% confidence interval.

The table demonstrates that although GPs feel that it is their responsibility and feel comfortable discussing anxiety disorders with families, inadequate training, as well as time restrictions and a lack of accessible treatment pose the biggest barriers for GPs in identification of childhood anxiety disorders. A lack of confidence, training, resources and time were the biggest barriers to management. GPs believed that specialist services were effective but waiting times posed the largest barrier to access and less than 10% of GPs felt that they had a relationship with these services. Stigma, language and culture issues were not seen as barriers to GPs' identification, management of referral.

For peer review only

Table 2. GP endorsement of facilitators and barriers to the identification, management and referral of childhood anxiety disorders

| **Item** | **% "agree" + "completely agree"** | **95% confidence interval** | | **% "disagree" + "completely disagree"** | **95% confidence interval** | |
|---|---|---|---|---|---|---|
| ***Identification of anxiety disorders in children under 12 years*** | | *Lower* | *Upper* | | *Lower* | *Upper* |
| *Facilitators* | | | | | | |
| Feel confident in ability | 53 | 50.78 | 57.06 | 12 | 9.91 | 13.99 |
| Believe training was adequate | 21 | 19.09 | 24.29 | 48 | 44.68 | 50.98 |
| Comfortable broaching idea of anxiety disorders with the child | 80 | 77.72 | 82.74 | 7 | 5.14 | 8.30 |
| Comfortable broaching idea of anxiety disorders with the family | 88 | 86.81 | 90.79 | 3 | 1.92 | 4.08 |
| Believe it is part of a GP's responsibility | 93 | 91.60 | 94.78 | 1 | 0.54 | 1.93 |
| *Endorsement of barriers* | **% "quite a lot" + "very much"** | | | **% "a little" + "not at all"** | | |
| Limitations in children's communication abilities | 21 | 19.05 | 24.24 | 33 | 29.93 | 35.84 |
| Misinformation from parents | 27 | 24.06 | 29.65 | 35 | 32.52 | 38.55 |
| Concerns about stigmatising the child | 10 | 8.68 | 12.56 | 69 | 66.77 | 72.56 |
| Family concerns about stigma | 16 | 13.60 | 18.21 | 60 | 57.25 | 63.41 |
| Cultural barriers | 17 | 14.46 | 19.17 | 58 | 54.89 | 61.11 |
| Language barriers | 18 | 15.83 | 20.69 | 64 | 61.38 | 67.41 |
| Family reluctance to accept disorder | 20 | 17.73 | 22.80 | 43 | 39.80 | 46.03 |

For peer review only

| | | | | | | |
|---|---|---|---|---|---|---|
| Time restrictions | 67 | 63.91 | 69.84 | 14 | 12.33 | 1.677 |
| Lack of available/accessible treatment | 68 | 98.34 | 99.61 | 15 | 12.91 | 17.42 |
| Lack of effective treatment | 45 | 42.53 | 48.80 | 27 | 23.91 | 29.48 |
| ***Management of children under 12 years with anxiety disorders*** | **% "agree" + "completely agree"** | | | **% "disagree" + "completely disagree"** | | |
| *Facilitators* | | | | | | |
| Feel confident in ability | 13 | 11.32 | 15.64 | 51 | 48.71 | 55.02 |
| Believe training was adequate | 10 | 8.29 | 12.09 | 66 | 63.56 | 69.50 |
| Comfortable discussing management strategies with the family | 52 | 49.33 | 55.63 | 20 | 17.32 | 22.34 |
| Comfortable discussing management strategies with the child | 46 | 43.29 | 49.58 | 22 | 19.21 | 24.42 |
| Aware of resources to aid families | 24 | 21.54 | 26.95 | 75 | 73.05 | 78.46 |
| Believe having a relationship with a family is beneficial | 94 | 92.54 | 95.52 | <1 | 0.39 | 1.66 |
| Aware of local agencies to support children and their families | 65 | 63.06 | 69.03 | 17 | 15.09 | 19.89 |
| Believe it is part of a GP's role | 69 | 66.70 | 72.49 | 10 | 8.01 | 11.77 |
| *Endorsement of barriers* | **% "quite a lot" + "very much"** | | | **% "a little" + "not at all"** | | |
| Cultural barriers | 14 | 12.01 | 16.43 | 60 | 58.72 | 64.86 |
| Language barriers | 16 | 13.98 | 18.65 | 64 | 61.32 | 67.37 |
| Time restrictions | 72 | 70.15 | 75.76 | 11 | 9.68 | 13.73 |
| Family reluctance to accept the | 19 | 16.78 | 21.76 | 46 | 43.38 | 49.68 |



For peer review only - http://bmjopen.bmj.com/site/about/guidelines.xhtml

| | | | | | | |
|---|---|---|---|---|---|---|
| disorder | | | | | | |
| Limitations in children's communication abilities | 18 | 15.80 | 20.67 | 43 | 40.60 | 46.86 |
| Misinformation from parents | 18 | 15.55 | 20.40 | 50 | 47/78 | 54.09 |
| Concerns about stigmatising the child | 7 | 5.91 | 9.25 | 71 | 69.31 | 74.98 |
| Family concerns about stigma | 11 | 9.23 | 13.21 | 63 | 60.58 | 66.66 |
| ***Referral of children under 12 years with anxiety disorders*** | **% "agree" + "completely agree"** | | | **% "disagree" + "completely disagree"** | | |
| *Facilitators* | | | | | | |
| Long waiting times reduce the likelihood of making a referral | 50 | 47.37 | 53.66 | 35 | 32.65 | 38.68 |
| Parental pressure increases the likelihood of making a referral | 85 | 83.18 | 87.62 | 5 | 4.02 | 6.87 |
| Have a relationship with local specialist services | 6 | 4.30 | 7.24 | 79 | 76.49 | 81.62 |
| Believe that specialist services' interventions are effective | 78 | 75.63 | 80.83 | 4 | 3.22 | 5.84 |
| *Endorsement of barriers* | **% "quite a lot" + "very much"** | | | **% "a little" + "not at all"** | | |
| Long waiting times | 89 | 87.03 | 90.97 | 3 | 1.91 | 4.04 |
| Cultural barriers | 8 | 6.22 | 9.65 | 76 | 75.11 | 80.38 |
| Language barriers | 8 | 6.83 | 10.40 | 77 | 76.49 | 81.66 |
| Time restrictions | 30 | 28.26 | 34.14 | 51 | 49.40 | 55.73 |
| Family reluctance to accept the disorder | 12 | 10.47 | 14.68 | 62 | 60.68 | 66.78 |
| Limitations in children's communication abilities | 6 | 4.91 | 8.03 | 70 | 68.19 | 73.93 |
| Misinformation from parents | 9 | 7.83 | 11.59 | 66 | 64.11 | 70.06 |



For peer review only - http://bmjopen.bmj.com/site/about/guidelines.xhtml

| | | | | | | |
|---|---|---|---|---|---|---|
| Concerns about stigmatising the child | 6 | 4.55 | 7.57 | 78 | 76.94 | 82.06 |
| Family concerns about stigma | 8 | 6.71 | 10.24 | 73 | 71.80 | 77.32 |
| Lack of available/accessible treatment | 54 | 51.78 | 58.10 | 28 | 25.49 | 31.22 |

248

For peer review only

ii) What factors are associated with a GPs confidence identifying managing this disorder?
A: The regression model (see Table 3) was a significant fit overall ($\chi^2$ (1) = 16.7, p < .05). Being female was significantly associated with higher confidence identifying anxiety disorders in children, (Ex(B) = 1.4), as was being in a less deprived practice (Ex(B) = 1.1). GP psychiatric and paediatric training, as well as years of experience and parenthood did not have any impact on their confidence, nor did size or ethnicity of their practice population.

Table 3. Regression model of factors are associated with a GP's confidence identifying childhood anxiety disorders

| | B | Exp(B) | 95% C.I. for EXP(B) | |
|---|---|---|---|---|
| | | | Lower | Upper |
| *GP* | | | | |
| Gender | .352* | 1.423 | 1.090 | 1.856 |
| Psychiatry training | .147 | 1.158 | .889 | 1.510 |
| Paediatric training | .155 | 1.168 | .861 | 1.5834 |
| Years qualified | -.004 | .996 | .980 | 1.012 |
| Child over 5 years | .163 | 1.177 | .841 | 1.656 |
| *Practice* | | | | |
| No. of patients | .000 | 1.000 | 1.000 | 1.000 |
| SES (deprivation decile) | .062* | 1.064 | 1.006 | 1.125 |
| % non-white ethnic groups | .008 | 1.008 | .995 | 1.021 |
| Constant | .081 | .922 | | |

* Note. $R^2$ =. 01 (Hosmer & Lemeshow) .01 (Cox & Snell) .03 (Nagelkerke). Model $\chi 2(1) = 17.4$, p < .05.

(ii) What factors are associated with a GPs confidence managing this disorder?
B: The regression model (see Table 4) was significant overall ($\chi^2$(1) = 18.2, p < .05.). Having a child over the age of 5 was significantly associated with greater confidence managing anxiety disorders in children (Ex(B)=1.99), as was being in a less deprived practice (Ex(B)=1.1). GP psychiatric and paediatric training, as well as years of experience and gender did not have any impact on their confidence, nor did size or ethnicity of their practice population.

Table 4. Regression model of factors which are associated with a GP's confidence managing childhood anxiety disorders

| | B | Exp(B) | 95% C.I. for EXP(B) | |
|---|---|---|---|---|
| | | | Lower | Upper |
| *GP* | | | | |
| Gender | -.239 | .787 | .531 | 1.168 |


For peer review only - http://bmjopen.bmj.com/site/about/guidelines.xhtml

| | | | | |
|---|---|---|---|---|
| Psychiatry training | .273 | 1.314 | .887 | 1.947 |
| Paediatric training | .218 | 1.244 | .766 | 2.019 |
| Years qualified | .002 | 1.002 | .979 | 1.027 |
| Child over 5 years | .687* | 1.987 | 1.132 | 3.489 |
| *Practice* | | | | |
| No. of patients | .000 | 1.000 | 1.000 | 1.000 |
| SES (deprivation decile) | .090* | 1.094 | 1.004 | 1.193 |
| % non-white ethnic groups | .015 | 1.015 | .997 | 1.034 |
| Constant | -1.847 | .158 | | |

* Note. $R^2$ =. 05 (Hosmer & Lemeshow) .02 (Cox & Snell) .04 (Nagelkerke). Model $\chi 2(1) = 20.23$, p = .01.

For peer review only

## Discussion

*Summary*

Although most GPs believe that it is their responsibility to identify and manage anxiety disorders in children, only half of those surveyed felt confident identifying, and only 13% felt confident managing these disorders. These figures may not be surprising given that only 21% of GPs believed their training in identification was adequate, just 10% believed that their training in management of anxiety disorders was adequate, and the vast majority were not aware of any resources to provide families. Time restrictions also posed a barrier for both identification and management. Although 78% of GPs believed that specialist services' interventions were likely to be effective, long waiting times and lack of accessible treatments were overwhelmingly endorsed as a barrier to accessing specialist services and 79% felt that they did not have a relationship with such services. Free-text comments for this section highlighted that GPs felt CAMHS referral criteria were unclear, and often had very high thresholds. However, notably 85% indicated that referral was significantly increased by parental pressure, demonstrating the need for parents to be strong advocates for their children in order to access specialist services. Interestingly, although previous literature reported stigma surrounding mental health as a barrier for GPs, this was not the case for the GPs in this research.

Female GPs and those in less deprived practices were associated with more confidence identifying child anxiety disorders. Notably, GPs who had completed psychiatric or paediatric training were not associated with more confidence in identification, reinforcing the commonly reported view that the training received was inadequate. In terms of management, GPs in less deprived practices and those who were parents of a child over the age of five were associated with more confidence. This latter finding was particularly striking and was consistent with our previous qualitative work that suggested that GPs rely on their own personal experience, as their professional training was largely viewed as inadequate, to manage these disorders(28).

*Strengths and limitations*

One of the key strengths of the study is the inclusion of a large, nationally representative group of GPs. Another is that it used findings from a recent systematic review(23), qualitative study(28) and GP feedback to inform the development of the questionnaire, to ensure that the questions were meaningful to the GPs and that the most pertinent questions were asked. This appeared to have been successful as the free-text responses mainly reinforced the findings from the main questionnaire and little new information was added. Unlike many previous studies, the questionnaire distinguished between different stages of the primary care process (i.e. identification, management, referral to specialist services). This allows pinpointing of where issues are most problematic, for example, time restrictions were a barrier for identification and management, but much less so for referral. We can also see that GPs feel significantly more confident identifying anxiety disorders than managing them. Future studies would benefit from examining these stages at a more granular level, for example, to further understand the skills and


For peer review only - http://bmjopen.bmj.com/site/about/guidelines.xhtml

confidence necessary at different stages of the process e.g. identifying the problem, broaching the subject with the family/young person.

These strengths need to be considered in the light of various limitations. Efforts were made to recruit a number of GPs to pilot the survey, however only one responded, which is a limitation of the study. Although the sample had similar demographic characteristics to GPs across England and we recruited a substantial proportion of GPs (n= 443; 46%) who did not consider themselves 'research active', no data were available on those who chose not to participate and we cannot exclude the possibility of a self-selection bias influencing the findings. We also did not assess GPs' abilities to accurately identify anxiety disorders in children and cannot rule out the possibility that in some cases GPs answered questions in relation to mental health disorders other than anxiety. This survey involved GPs reporting on their own experiences, which may be influenced by poor recall and social desirability biases. Furthermore, as this survey is cross-sectional, conclusions cannot be drawn about causality. Most free text comments related to questions asked in the survey, however an additional area that GPs raised in the comments was the role of others in identification of anxiety disorders and for referrals to specialist services, in particular, schools and health visitors. A final limitation of this study is the lack of distinction between anxiety disorders, which are not a homogenous group, as well as the fact that we focused on anxiety disorders as a distinct condition, despite the fact that they are often co-morbid with other psychological and physical health disorders. This may limit the generalisability of this study.

*Comparison with previous research*

The specific focus on anxiety disorders is a further strength of this research given the specific challenges faced with this condition, and this contrasts with much of the previous work in this area which does not distinguish between mental health disorders. Many of the findings in this study echo previous qualitative findings(24, 28), such as the positive impact of being a parent on confidence managing child anxiety disorders and issues associated with long waiting times and poor access to specialist services. It also reinforced the idea that GPs working in more deprived areas may experience particular difficulties potentially due to more complex family situations. Future studies would benefit from a more in-depth examination into the association between deprivation and GP confidence in identification and management. However, although some of the GPs interviewed in the previous research seemed to have some uncertainty regarding their role in the identification and management of this condition, it appeared that the majority of GPs surveyed believed that this is their responsibility, at least in part. This research also reinforced a recent stem4 report which found that GPs had serious concerns about accessing specialist help for young people with mental health disorders(32). The issue of poor access to services and long waiting times are common across the mental health literature, but this study highlights a number of novel findings, such as the widespread lack of confidence in management of childhood


For peer review only - http://bmjopen.bmj.com/site/about/guidelines.xhtml

anxiety disorders, and the stronger role of personal experience of being a parent than access to relevant specialist training in GP confidence managing childhood anxiety disorders.

*Implications for clinicians, policy-makers and researchers*

The focus of this research was to investigate barriers, from a GP's perspective, however in order to fully understand how to increase access to appropriate support for childhood anxiety disorders, future studies could seek to capture objective data (e.g. referral data), in order to corroborate the questionnaire findings. This would be useful to understand whether self-report correlates with actual behavior. Furthermore, providing GPs with vignettes describing children with and without anxiety disorders may be useful to determine whether a GP's perception of their confidence is related to their actual identification abilities.

This research strongly suggests that GPs' current training in childhood anxiety disorders is inadequate, and highlights the need to place increased emphasis on training, both in medical school training and beyond, to boost confidence and develop skills. This reinforces previous qualitative findings which highlight that GPs believed that their training in mental health problems had been inadequate, and focused on adults to the detriment of children and young people(33). This training should be meaningful for GPs and firmly grounded in the context of primary care, as well as focused specifically on child and adolescent populations. This supports recommendations from the RCGP that GPs should receive specialist-led training in child health and mental health problems(34). It is encouraging that the majority of GPs surveyed see identifying and managing this condition as part of their role, in addition to other organisations (e.g. schools), but it is possible that a lack of confidence and comfort with the topic matter prevents GPs from fully engaging with a condition(35). In addition to further training, previous qualitative research suggests that increased contact and collaboration with specialist services would increase GPs confidence in this area(33). Furthermore, as with previous studies in this area, difficulty accessing specialist services in a timely manner poses a significant barrier for GPs who commonly used the free-text comments to express the view that CAMHS' referral thresholds are prohibitively high, and service inclusion criteria are unclear. Increasing GPs confidence in this area, as well as providing them with more effective resources, may encourage and enable GPs to manage many cases in primary care and only refer severe or complex cases, thus alleviating pressure on specialist services.

For peer review only

**Conclusion**

Failing to intervene effectively with childhood anxiety disorders brings negative implications for both individual children and families(6) and increased costs for society(8). GPs consider identification and management of childhood anxiety disorders to be within their role however they face a number of barriers and lack confidence in their abilities. Increased GP training in identification and management of child anxiety disorders may help to strengthen GPs'



For peer review only - http://bmjopen.bmj.com/site/about/guidelines.xhtml

confidence and ability to effectively support children under the age of 12 with anxiety disorders. Furthermore, the findings suggest that GPs would benefit from increased access to ongoing training and appropriate resources to support identification and early management and to facilitate better communication around access to specialist services for children with anxiety disorders.

**Acknowledgements:** We acknowledges the support of the following National Institute for Health Research Clinical Research Networks (NIHR CRN): Thames Valley & South Midlands, East Midlands, Eastern, North West London, South London, North East and North Cumbria, South West Peninsula, West Midlands, Yorkshire & Humber, North Thames, Wessex.

For peer review only

# References


1. Kessler RC, Ruscio AM, Shear K, Wittchen H-U. Epidemiology of anxiety disorders. Behavioral neurobiology of anxiety and its treatment: Springer; 2009. p. 21-35.
2. Kessler R, C.;, Berglund P, Demler O, Jin R, Merikangas KR, Walters EE. Lifetime Prevalence and Age-of-Onset Distributions of DSM-IV Disorders in the National Comorbidity Survey Replication. JAMA Psychiatry. 2005;62(6):593.
3. Lawrence D, Hafekost J, Johnson SE, Saw S, Buckingham WJ, Sawyer MG, et al. Key findings from the second Australian Child and Adolescent Survey of Mental Health and Wellbeing. Aust N Z J Psychiatry. 2016;50(9):876-86.
4. Merikangas KR, He JP, Burstein M, Swanson SA, Avenevoli S, Cui L, et al. Lifetime prevalence of mental disorders in U.S. adolescents: results from the National Comorbidity Survey Replication--Adolescent Supplement (NCS-A). J Am Acad Child Adolesc Psychiatry. 2010;49(10):980-9.
5. Polanczyk GV, Salum GA, Sugaya LS, Caye A, Rohde LA. Annual Research Review: A meta-analysis of the worldwide prevalence of mental disorders in children and adolescents. J Child Psychol Psychiatry. 2015;56(3):345-65.
6. Woodward LJ, Fergusson DM. Life course outcomes of young people with anxiety disorders in adolescence. J Am Acad Child Adolesc Psychiatry. 2001;40(9):1086-93.
7. Knapp M, Snell T, Healey A, Guglani S, Evans-Lacko S, Fernandez JL, et al. How do child and adolescent mental health problems influence public sector costs? Interindividual variations in a nationally representative British sample. J Child Psychol Psychiatry. 2015;56(6):667-76.
8. Fineberg NA, Haddad PM, Carpenter L, Gannon B, Sharpe R, Young AH, et al. The size, burden and cost of disorders of the brain in the UK. J Psychopharmacol. 2013;27(9):761-70.
9. Kassebaum NJ, Arora M, Barber RM, Bhutta ZA, Brown J, Carter A, et al. Global, regional, and national disability-adjusted life-years (DALYs) for 315 diseases and injuries and healthy life expectancy (HALE), 1990-2015: a systematic analysis for the Global Burden of Disease Study 2015. The Lancet. 2016;388(10053):1603.
10. Craske MG, Stein MB. Anxiety. The Lancet. 2016;388(10063):3 048-4059.
11. James AC, James G, Cowdrey FA, Soler A, Choke A. Cognitive behavioural therapy for anxiety disorders in children and adolescents. Cochrane Database Syst Rev. 2013(6):CD004690.
12. Zhen W, H. WSP, Leslie S, Wigdan F, S. MA, Mouaz A, et al. Comparative Effectiveness and Safety of Cognitive Behavioral Therapy and Pharmacotherapy for Childhood Anxiety Disorders A Systematic Review and Meta-analysis. JAMA Paediatrics. 2017.
13. Merikangas KR, He JP, Burstein M, Swendsen J, Avenevoli S, Case B, et al. Service utilization for lifetime mental disorders in U.S. adolescents: results of the National Comorbidity Survey-Adolescent Supplement (NCS-A). J Am Acad Child Adolesc Psychiatry. 2011;50(1):32-45.
14. Essau CA. Frequency and patterns of mental health services utilization among adolescents with anxiety and depressive disorders. Depress Anxiety. 2005;22(3):130-7.
15. Hippisley-Cox, J, Y. V. Trends in consultation rates in general practice 1995/1996 to 2008/2009: analysis of the QResearch database. . . 2009.
16. Charles J, Harrison CM, Britt H. Management of children's psychological problems in general practice 1970-1971, 1990-1991 and 2008-2009. Australian & New Zealand Journal of Psychiatry. 2011;45(11):976-84.




For peer review only - http://bmjopen.bmj.com/site/about/guidelines.xhtml

17. Kelleher KJ, McInerny TK, Gardner WP, Childs GE, Wasserman RC. Increasing identification of psychosocial problems: 1979-1996. Pediatrics. 2000;105(6):1313-21.

18. Roberts JH, Bernard PM. 'Can he have the test for bipolar, doctor? His dad's got it': Exploring the potential of general practitioners to work with children and young people presenting in primary care with common mental health problems - A clinical initiative. Mental Health in Family Medicine. 2012;9(2):115-23.

19. Committee oH. The future of pediatrics: Mental health competencies for pediatric primary care. Pediatrics. 2009;124(1):410-21.

20. Department for Children SaF. *Early intervention: securing good outcomes for all children and young people*Early intervention: securing good outcomes for all children and young people. London: *Department for Children, Schools and Families*Department for Children, Schools and Families; 2010.

21. Roberts JH, Crosland A, Fulton J. "I think this is maybe our Achilles heel..." exploring GPs' responses to young people presenting with emotional distress in general practice: a qualitative study. BMJ Open. 2013;3(9):e002927.

22. Chien A, Coker T, Choi L, Slora E, Bodnar P, Weiley V, et al. What do pediatric primary care providers think are important research questions? A perspective from PROS providers. Ambul Pediatr. 2006;6(6):352-5.

23. O'Brien D, Harvey K, Howse J, Reardon T, Creswell C. Barriers to managing child and adolescent mental health problems: a systematic review of primary care practitioners' perceptions. Br J Gen Pract. 2016:bjgpoct-2016-66-651-harvey-fl-p.

24. Hinrichs S, Owens M, Dunn V, Goodyer I. General practitioner experience and perception of Child and Adolescent Mental Health Services (CAMHS) care pathways: a multimethod research study. BMJ Open. 2012;2(6).

25. Sayal K, Taylor E. Detection of child mental health disorders by general practitioners. British Journal of General Practice. 2004;54(502):348-52.

26. Burke DA, Koot HM, de Wilde A, Begeer S. Influence of Child Factors on Health-Care Professionals' Recognition of Common Childhood Mental-Health Problems. Journal of child and family studies. 2016;25(10):3083-96.

27. Jacobs CO, Loades ME. An investigation into GPs' perceptions of children's mental health problems. Child and Adolescent Mental Health. 2016;21(2):90-5.

28. O'Brien D, Harvey K, Reardon T, Young B, Creswell C. General Practitioners' Experiences of Children with Anxiety
Disorders in Primary Care: a Qualitative Study. British Journal of General Practice. 2017.

29. Digital GWTN. General and Personal Medical Services,
England September 2015 - March 2016. Health and Social Care Information Centre; 2016.

30. Peduzzi P, Concato J, Kemper E, Holford TR, Feinstein AR. A simulation study of the number of events per variable in logistic regression analysis. Journal of clinical epidemiology. 1996;49(12):1373-9.

31. Corp I. IBM SPSS Statistics for Windows, Version 22.0. Armonk, NY: IBM Corp; 2013.

32. stem4. "A Time Bomb Waiting to Explode" A Survey of GPs' Concerns about Mental Health Services for Children and Young People. London; 2016.

33. O'Brien D, Harvey K, Reardon T, Young B, Creswell C. General Practitioners' Experiences of Children with Anxiety

For peer review only

494 Disorders in Primary Care: a Qualitative Study. British Journal of General Practice. 2017.
495 34. Practice RCoG. Enhanced and extended GP training progress update. London; 2013.
496 35. Salmon P, Peters S, Clifford R, Iredale W, Gask L, Rogers A, et al. Why do general practitioners decline training to improve management of medically unexplained symptoms? Journal of General Internal Medicine. 2007;22(5):565-71.
499
500

For peer review only

**Author Statement:** All three authors (KH, DOB, CC) were involved in the design of this study. Participant recruitment and analysis was conducted by DOB. The main draft of the manuscript was written by DOB with input by KH and CC.

**Data sharing statement:** Full dataset and statistical codes are available from the corresponding author. 'Data is currently being uploading to the University of Reading research data archive and a link will be provided prior to publication'

For peer review only

# CADPC
## Consent page

**Children with Anxiety Disorders in Primary Care: the views of General Practitioners II**

We invite you to take part in a National Institute of Health Research funded study which focuses on management of childhood anxiety disorders (children under 12 years) by General Practitioners in England. This study explores GPs' experiences of identification, management and referral of these disorders.

**Please note, while some of these questions may seem repetitive, consider whether they are being asked in relation to either identification, management or referral.** Your responses will help inform practice guidelines for children with anxiety disorders.

**Taking part in this study will involve completing a survey, which should take 10-15 minutes to complete. This is open to as many GPs per surgery as desired.**

As a reimbursement for taking part in the study you (or a nominated member of your practice) will have the opportunity to take part in a Webinar about identification and management of childhood anxiety disorders. This module may be used to contribute towards Continued Professional Development points and a certificate of attendance outlining your involvement in important mental health research will be provided. You will also be entered into a prize draw to win one of two £100 Amazon vouchers.

We are happy to answer queries about the study, please contact the researchers for further information:

Doireann O'Brien, B.A., M.Sc.
Email: doireann.obrien@pgr.reading.ac.uk

Professor Cathy Creswell
Tel: +44 (0)118 378 6798

Further details of the study are available as an information sheet. Please find information sheet enclosed.

**All items of the consent form, below, must be initialled to indicate consent.**

For peer review only

I can confirm that I have read and understand the information sheet, have had the opportunity to ask questions and, if applicable, have had these questions answered satisfactorily ⓘ *Required* ☐

I understand that my participation is voluntary, that I can withdraw my consent at any time, without giving a reason and without my professional role being compromised ⓘ *Required* ☐

I understand that the answers to my questions will be stored in a secure location, but will bear no identifying information. I also understand that the data will be kept for 12 months and after this time they will be destroyed ⓘ *Required* ☐

I understand that, should I lose the capacity to consent to be in the study, the research centre will retain any information collected prior to this point ⓘ *Required* ☐

I understand that relevant sections of my data collected during the study, may be looked at by individuals from the University of Reading, from regulatory authorities or from the NHS Trust, where it is relevant to my taking part in this research. I give permission for these individuals to have access to my records ⓘ *Required* ☐

I agree to take part in the study ⓘ *Required* ☐

For peer review only

# SURVEY

| | |
|---|---|
| Age: *(please circle)* 23-35 35-45 45-55 55-65 65+ | |
| Identify my gender as: *(please circle)* MALE FEMALE OTHER | |
| Psychiatric rotation completed as part of GP training: *(please circle)* YES NO | |
| Paediatric rotation completed as part of GP training: *(please circle)* YES NO | |
| Year qualified as a GP: | |
| Practice Name: | |
| Practice Postcode: | |
| Years employed in current Trust: | |
| Would you consider yourself research active? *(please circle)* YES NO | |
| Are you a parent or guardian? *(please circle)* YES NO | |
| If so, what ages are your children: | |
| Compared to other GPs in your practice, do you see more or fewer child patients? *(please circle)* YES NO SIMILAR AMOUNT | |

For peer review only

PLEASE NOTE: **ALL QUESTIONS ARE IN RELATION TO ANXIETY IN CHILDREN AGED 12 & UNDER (i.e. primary school age and below)**

For each item identified below, circle the number to the right that best fits your judgment of its quality.
Use the rating scale to select the number.

| **Survey Item** | **Scale***<br>**1 = completely disagree**<br>**2 = disagree**<br>**3 = neutral**<br>**5 = agree**<br>**5 = completely agree**<br><br>*unless otherwise specified | | | | |
|---|---|---|---|---|---|
| **Identification**<br>*These questions are in relation to initially identifying the presence of an Anxiety Disorder in a child* | | | | | |
| 1. How common are anxiety disorders in children in your experience? | 1 in 3 | 1 in 10 | 1 in 15 | 1 in 20 | 1 in 40 |
| 2. How often do you see anxiety disorders in your practice? | | | | | |
| 3. I believe that anxiety disorders are prevalent in children under 12 | 1 | 2 | 3 | 4 | 5 |
| 4. I am confident in my ability to recognise anxiety disorders in children | 1 | 2 | 3 | 4 | 5 |
| 5. I am regularly looking out for anxiety disorders in children | 1 | 2 | 3 | 4 | 5 |
| 6. My training adequately equipped me with the skills to identify anxiety disorders in children | 1 | 2 | 3 | 4 | 5 |
| 7. I use screening tools to aid identification of anxiety disorders in children (Screening tools refer to validated measures used to aid identification of a suspected anxiety disorder) | Yes | | | No | |
| a. *If yes:* I find screening tools useful for the identification of anxiety disorders in children | 1 | 2 | 3 | 4 | 5 |
| b. *If no:* I would find a brief screening tool useful for identification of anxiety disorders in children | 1 | 2 | 3 | 4 | 5 |
| 8. I am comfortable broaching the subject of a potential anxiety disorder with: | | | | | |
| a. the child | 1 | 2 | 3 | 4 | 5 |
| b. the family | 1 | 2 | 3 | 4 | 5 |
| 9. I believe that it is part of my responsibility in my role as a GP to identify anxiety disorders in children | 1 | 2 | 3 | 4 | 5 |
| 10. Other agencies are responsible for identifying childhood anxiety disorders | 1 | 2 | 3 | 4 | 5 |
| a. *If yes*: Name agencies here | | | | | |

| 11. To what extent do the following factors get in the way of your ability to accurately identify anxiety disorders in children: | For the following:<br>1 = Not at all<br>2= A little<br>3 = Somewhat<br>4 = Quite a lot<br>5 = Very much | | | | |
|---|---|---|---|---|---|
| a. Limitations in children's communication abilities | 1 | 2 | 3 | 4 | 5 |
| b. Misinformation from parents | 1 | 2 | 3 | 4 | 5 |
| c. My concerns about stigmatising the child | 1 | 2 | 3 | 4 | 5 |
| d. Family concerns about stigma | 1 | 2 | 3 | 4 | 5 |
| e. Cultural barriers | 1 | 2 | 3 | 4 | 5 |
| f. Language barriers | 1 | 2 | 3 | 4 | 5 |
| g. Time restrictions | 1 | 2 | 3 | 4 | 5 |
| h. Family reluctance to accept disorder | 1 | 2 | 3 | 4 | 5 |
| i. Lack of training | 1 | 2 | 3 | 4 | 5 |
| j. Lack of experience | 1 | 2 | 3 | 4 | 5 |
| k. Lack of knowledge | 1 | 2 | 3 | 4 | 5 |
| l. Lack of available/accessible treatment | 1 | 2 | 3 | 4 | 5 |
| m. Lack of effective treatment | 1 | 2 | 3 | 4 | 5 |
| Please add any further barriers to identification here if necessary | | | | | |
| n. | 1 | 2 | 3 | 4 | 5 |
| o. | 1 | 2 | 3 | 4 | 5 |
| p. | 1 | 2 | 3 | 4 | 5 |
| q. | | | | | |
| r. | | | | | |

| Survey Item | Scale*<br>1 = completely disagree<br>2 = disagree<br>3 = neutral<br>5 = agree<br>5 = completely agree<br><br>*unless otherwise specified |
|---|---|

**Management:**

*These questions are in relation to your ongoing management of a childhood Anxiety Disorders, either prior to making a referral or in the absence of a referral*

For peer review only

| | | | | | |
|---|---|---|---|---|---|
| 12. I believe that I have had adequate training to manage anxiety disorders in children | 1 | 2 | 3 | 4 | 5 |
| 13. I have adequate skills to manage anxiety disorders in children | 1 | 2 | 3 | 4 | 5 |
| 14. I feel confident managing anxiety disorders in children | 1 | 2 | 3 | 4 | 5 |
| 15. I am comfortable discussing management strategies for anxiety disorders with: | | | | | |
| a. the child | 1 | 2 | 3 | 4 | 5 |
| b. the family | 1 | 2 | 3 | 4 | 5 |
| 16. I provide families with management tips & strategies for managing anxiety disorders in children | 1 | 2 | 3 | 4 | 5 |
| 17. I am aware of resources such as books/websites/apps to aid families in the management of anxiety disorders in children | Yes | | | No | |
| a. *If yes:* I use resources such as books/websites/apps in the management of childhood anxiety disorders | 1 | 2 | 3 | 4 | 5 |
| b. *If yes:* Please state which ones | | | | | |
| c. *If no:* I would use resources such as books/websites/apps in the management of childhood anxiety disorders | 1 | 2 | 3 | 4 | 5 |
| 18. Are you aware of any online/apps resources for parents concerned about their child's anxiety? | YES | | | NO | |
| a. *If Yes*: please state which ones | | | | | |
| b. *If Yes:* I have sign-posted parents concerned about their child's anxiety to these | 1 | 2 | 3 | 4 | 5 |
| c. *If No:* If I were aware of online/app resources, I would signpost parents to these if they were concerned about their child's anxiety | 1 | 2 | 3 | 4 | 5 |
| 19. It would be important for an online/app resource for childhood anxiety to have an evidence-base in order for me to recommend it | 1 | 2 | 3 | 4 | 5 |
| 20. It would be important to have NHS accreditation of the online/resources for childhood anxiety be in order for me to recommend it | 1 | 2 | 3 | 4 | 5 |
| 21. Having a relationship with a family aids me in the management of anxiety disorders | 1 | 2 | 3 | 4 | 5 |
| 22. I involve the child's school in the management of anxiety disorders | 1 | 2 | 3 | 4 | 5 |
| a. *If yes:* I find this helpful | 1 | 2 | 3 | 4 | 5 |
| 23. I am aware of local agencies available to support children with anxiety disorders and their families | 1 | 2 | 3 | 4 | 5 |
| 24. I believe it is part of my role as a GP to manage anxiety disorders in children | 1 | 2 | 3 | 4 | 5 |
| 25. Other agencies are responsible for managing childhood anxiety disorders | 1 | 2 | 3 | 4 | 5 |
| a. Add other agencies here if applicable | | | | | |

For peer review only - http://bmjopen.bmj.com/site/about/guidelines.xhtml

| 26. To what extent do the following factors get in the way of your ability to effectively manage anxiety disorders in children: | **For the following:**<br>**1 = Not at all**<br>**2= A little**<br>**3 = Somewhat**<br>**4 = Quite a lot**<br>**5 = Very much** | | | | |
|---|---|---|---|---|---|
| a. Cultural barriers | 1 | 2 | 3 | 4 | 5 |
| b. Language barriers | 1 | 2 | 3 | 4 | 5 |
| c. Time restrictions | 1 | 2 | 3 | 4 | 5 |
| d. Family reluctance to accept the disorder | 1 | 2 | 3 | 4 | 5 |
| e. Limitations in children's communication abilities | 1 | 2 | 3 | 4 | 5 |
| f. Misinformation from parents | 1 | 2 | 3 | 4 | 5 |
| g. My concerns about stigmatising the child | 1 | 2 | 3 | 4 | 5 |
| h. Family concerns about stigma | 1 | 2 | 3 | 4 | 5 |
| i. Lack of training | 1 | 2 | 3 | 4 | 5 |
| j. Lack of experience | 1 | 2 | 3 | 4 | 5 |
| k. Lack of knowledge | 1 | 2 | 3 | 4 | 5 |
| Please add any further barriers to management below if necessary | | | | | |
| l. | 1 | 2 | 3 | 4 | 5 |
| m. | 1 | 2 | 3 | 4 | 5 |
| n. | 1 | 2 | 3 | 4 | 5 |

| **Survey Item** | **Scale***<br>**1 = completely disagree**<br>**2 = disagree**<br>**3 = neutral**<br>**5 = agree**<br>**5 = completely agree**<br><br>*unless otherwise specified | | | | |
|---|---|---|---|---|---|
| **Referral**<br>*These questions are in relation to a referral of a childhood Anxiety Disorder to a specialist service (such as Child & Adolescent Mental Health Services)* | | | | | |
| 27. How often in the last 5 years have you referred a child to specialist services for an anxiety disorder? | 0 times | 0-5 times | 5-10 times | 10-15 times | 15+ times |
| 28. Parental pressure increases the likelihood that I would refer a child with an anxiety disorder to specialist services | 1 | 2 | 3 | 4 | 5 |
| 29. anxiety disorders in children will generally resolve itself without treatment | 1 | 2 | 3 | 4 | 5 |
| 30. I seek specialist advice prior to making a referral of a childhood anxiety disorder | 1 | 2 | 3 | 4 | 5 |

| | | | | | |
|---|---|---|---|---|---|
| a. *If yes:* I find this helpful | 1 | 2 | 3 | 4 | 5 |
| b. *If no:* I think that I would find this helpful | 1 | 2 | 3 | 4 | 5 |
| 31. I experience long waiting times for referral for a childhood anxiety disorders | 1 | 2 | 3 | 4 | 5 |
| a. *If yes:* Long waiting times reduce the likelihood that I would make a referral to specialist services for childhood anxiety disorders | 1 | 2 | 3 | 4 | 5 |
| 32. I feel like I have a relationship with specialist services for childhood anxiety disorders | 1 | 2 | 3 | 4 | 5 |
| a. *If yes:* This is helpful when making a referral | 1 | 2 | 3 | 4 | 5 |
| b. *If no:* I think this would this be helpful when making a referral | 1 | 2 | 3 | 4 | 5 |
| 33. I think that specialist services' interventions for childhood anxiety disorders are likely to be effective | 1 | 2 | 3 | 4 | 5 |
| a. *If no:* This reduces the likelihood that I will make a referral | 1 | 2 | 3 | 4 | 5 |
| 34. I see my role mostly as a sign-poster/referrer for childhood anxiety disorders | 1 | 2 | 3 | 4 | 5 |
| 35. Other agencies are responsible for referring childhood anxiety disorders | 1 | 2 | 3 | 4 | 5 |
| a. *If yes:* Name other agencies here | | | | | |
| 36. To what extent do the following factors get in the way of your ability to refer children with anxiety disorders to specialist services: | **For the following:**<br>**1 = Not at all**<br>**2= A little**<br>**3 = Somewhat**<br>**4 = Quite a lot**<br>**5 = Very much** | | | | |
| a. Cultural barriers | 1 | 2 | 3 | 4 | 5 |
| b. Language barriers | 1 | 2 | 3 | 4 | 5 |
| c. Time restrictions | 1 | 2 | 3 | 4 | 5 |
| d. Family reluctance to accept the disorder | 1 | 2 | 3 | 4 | 5 |
| e. Waiting times for specialist services | 1 | 2 | 3 | 4 | 5 |
| f. Limitations in children's communication abilities | 1 | 2 | 3 | 4 | 5 |
| g. Misinformation from parents | 1 | 2 | 3 | 4 | 5 |
| h. My concerns about stigmatising the child | 1 | 2 | 3 | 4 | 5 |
| i. Family concerns about stigma | 1 | 2 | 3 | 4 | 5 |
| j. Lack of training | 1 | 2 | 3 | 4 | 5 |
| k. Lack of experience | 1 | 2 | 3 | 4 | 5 |
| l. Lack of knowledge | 1 | 2 | 3 | 4 | 5 |
| m. Lack of available/accessible treatment | 1 | 2 | 3 | 4 | 5 |

For peer review only

| | | | | | |
|---|---|---|---|---|---|
| n. Lack of effective treatment | 1 | 2 | 3 | 4 | 5 |
| Please add any further barriers to referral below if necessary | | | | | |
| o. | 1 | 2 | 3 | 4 | 5 |
| p. | 1 | 2 | 3 | 4 | 5 |
| q. | 1 | 2 | 3 | 4 | 5 |
| Please use this box to add any additional comments you may have | | | | | |

For peer review only

**STROBE 2007 (v4) Statement—Checklist of items that should be included in reports of *cross-sectional studies***

| Section/Topic | Item # | Recommendation | Reported on page # |
|---|---|---|---|
| **Title and abstract** | 1 | (*a*) Indicate the study's design with a commonly used term in the title or the abstract | 1 |
| | | (*b*) Provide in the abstract an informative and balanced summary of what was done and what was found | 2 |
| **Introduction** | | | |
| Background/rationale | 2 | Explain the scientific background and rationale for the investigation being reported | 4-5 |
| Objectives | 3 | State specific objectives, including any prespecified hypotheses | 4-5 |
| **Methods** | | | |
| Study design | 4 | Present key elements of study design early in the paper | 6-7 |
| Setting | 5 | Describe the setting, locations, and relevant dates, including periods of recruitment, exposure, follow-up, and data collection | 6-7 |
| Participants | 6 | (*a*) Give the eligibility criteria, and the sources and methods of selection of participants | 6-7 |
| Variables | 7 | Clearly define all outcomes, exposures, predictors, potential confounders, and effect modifiers. Give diagnostic criteria, if applicable | 6-7 |
| Data sources/ measurement | 8* | For each variable of interest, give sources of data and details of methods of assessment (measurement). Describe comparability of assessment methods if there is more than one group | 6-7 |
| Bias | 9 | Describe any efforts to address potential sources of bias | |
| Study size | 10 | Explain how the study size was arrived at | 6 |
| Quantitative variables | 11 | Explain how quantitative variables were handled in the analyses. If applicable, describe which groupings were chosen and why | 6-7 |
| Statistical methods | 12 | (*a*) Describe all statistical methods, including those used to control for confounding | 6-7 |
| | | (*b*) Describe any methods used to examine subgroups and interactions | 6-7 |
| | | (*c*) Explain how missing data were addressed | 6-7 |
| | | (*d*) If applicable, describe analytical methods taking account of sampling strategy | 6-7 |
| | | (*e*) Describe any sensitivity analyses | |
| **Results** | | | |

For peer review only

| | | | |
|---|---|---|---|
| Participants | 13* | (a) Report numbers of individuals at each stage of study—eg numbers potentially eligible, examined for eligibility, confirmed eligible, included in the study, completing follow-up, and analysed | 8 |
| | | (b) Give reasons for non-participation at each stage | N/a |
| | | (c) Consider use of a flow diagram | N/a |
| Descriptive data | 14* | (a) Give characteristics of study participants (eg demographic, clinical, social) and information on exposures and potential confounders | 8 |
| | | (b) Indicate number of participants with missing data for each variable of interest | 8 |
| Outcome data | 15* | Report numbers of outcome events or summary measures | 8 |
| Main results | 16 | (*a*) Give unadjusted estimates and, if applicable, confounder-adjusted estimates and their precision (eg, 95% confidence interval). Make clear which confounders were adjusted for and why they were included | 8 |
| | | (*b*) Report category boundaries when continuous variables were categorized | N/A |
| | | (*c*) If relevant, consider translating estimates of relative risk into absolute risk for a meaningful time period | N/A |
| Other analyses | 17 | Report other analyses done—eg analyses of subgroups and interactions, and sensitivity analyses | 8 |
| **Discussion** | | | |
| Key results | 18 | Summarise key results with reference to study objectives | 9 |
| Limitations | 19 | Discuss limitations of the study, taking into account sources of potential bias or imprecision. Discuss both direction and magnitude of any potential bias | 9 |
| Interpretation | 20 | Give a cautious overall interpretation of results considering objectives, limitations, multiplicity of analyses, results from similar studies, and other relevant evidence | 10 |
| Generalisability | 21 | Discuss the generalisability (external validity) of the study results | 10-11 |
| **Other information** | | | |
| Funding | 22 | Give the source of funding and the role of the funders for the present study and, if applicable, for the original study on which the present article is based | 2 |

*Give information separately for cases and controls in case-control studies and, if applicable, for exposed and unexposed groups in cohort and cross-sectional studies.

**Note:** An Explanation and Elaboration article discusses each checklist item and gives methodological background and published examples of transparent reporting. The STROBE checklist is best used in conjunction with this article (freely available on the Web sites of PLoS Medicine at http://www.plosmedicine.org/, Annals of Internal Medicine at http://www.annals.org/, and Epidemiology at http://www.epidem.com/). Information on the STROBE Initiative is available at www.strobe-statement.org.

For peer review only

BMJ Open

# Barriers and facilitators to identification and management of childhood anxiety disorders in primary care: a survey of General Practitioners in England

| | |
|---|---|
| Journal: | *BMJ Open* |
| Manuscript ID | bmjopen-2018-023876.R2 |
| Article Type: | Research |
| Date Submitted by the Author: | 14-Feb-2019 |
| Complete List of Authors: | O'Brien, Doireann; University of Reading, Psychology & Clinical Language Sciences<br>Harvey, Kate; University of Reading, Psychology<br>Creswell, Cathy; University of Reading, Psychology & Clinical Language Sciences |
| <b>Primary Subject Heading</b>: | Mental health |
| Secondary Subject Heading: | Paediatrics, General practice / Family practice |
| Keywords: | Anxiety disorders < PSYCHIATRY, PRIMARY CARE, Child & adolescent psychiatry < PSYCHIATRY |
| | |

SCHOLARONE™
Manuscripts

# Barriers and facilitators to identification and management of childhood anxiety disorders in primary care: a survey of General Practitioners in England

Doireann O'Brien,[1] Kate Harvey,[1*], Cathy Creswell [1]

[1] School of Psychology and Clinical Language Sciences, University of Reading, UK

*Correspondence to Associate Professor Kate Harvey (k.n.harvey@reading.ac.uk)

For peer review only

## Abstract

**Objectives:** Although anxiety disorders are the most common emotional disorders in childhood and are associated with a broad range of negative outcomes, only a minority of affected children receive professional support. In the UK, General Practitioners(GPs) are seen as "gate-keepers" to mental health services. The aim of this study was to examine the extent to which GPs experience barriers and facilitators to identifying, managing and accessing specialist services for these disorders, as well as factors associated with GPs' confidence.

**Design & setting**: Cross-sectional, self-report questionnaire in primary care, addressing identification, management and access to specialist services for children(under 12 years) with anxiety disorders

**Participants:** 971 GPs in England

**Primary outcomes:** The primary outcomes for this research was the extent to which GPs felt confident a)identifying and b)managing anxiety disorders in children.

**Results:** Only 51% and 13% of GPs felt confident identifying and managing child anxiety disorders respectively. A minority believed that their training in identification(21%) and management(10%) was adequate. Time restrictions inhibited identification and management, and long waiting times was a barrier to accessing specialist services. Being female(Ex(B)=1.4,95%CI 1.1-1.9) and being in a less deprived practice(Ex(B)=1.1,95%CI 1-1.1) was associated with higher confidence identifying childhood anxiety disorders. Being a parent of a child over the age of 5(Ex(B)=2,95%CI 1.1-3.5) and being in a less deprived practice(Ex(B)=1.1,95%CI 1-1.2) was associated with higher confidence in management. Receipt of psychiatric or paediatric training was not significantly associated with GP confidence.

**Conclusions**: GPs believe they have a role in identifying and managing childhood anxiety disorders, however their confidence appears to be related to their personal experience and the context in which they work, rather than their training, highlighting the need to strengthen GP training and facilitate access to resources and services to enable them to support children with these common but debilitating conditions.

**Funding:** This work was supported by grant a National Institute for Health Research 'Research Professorship' to CC (NIHR-RP-2014-04-018). The views expressed are those of the authors and not necessarily those of the NHS, the NIHR or the Department of Health.

**Competing Interests:** None
**Word count:** 3620

## Strengths and limitations of this study

- A key strength of the study is the inclusion of a large, nationally representative group of GPs.
- Findings from a recent systematic review, qualitative study and GP feedback all informed the development of the questionnaire ensured that the most pertinent questions were asked and that the questions were meaningful to the GPs.
- The questionnaire distinguished between different stages of the primary care process which allowed pinpointing of at which stage issues are most problematic.
- No data was available for GPs who chose not to participate and the possibility of self-selection biases influencing the findings cannot be excluded.
- GPs' abilities to accurately identify anxiety disorders in children was not assessed and the possibility that, in some cases, GPs answered questions in relation to mental health disorders other than anxiety cannot be ruled out.

**Keywords:** Primary Health Care, General Practice, Child Mental Disorders, Access to Health Care, Barriers, Anxiety Disorders

For peer review only



For peer review only - http://bmjopen.bmj.com/site/about/guidelines.xhtml

## Introduction

Anxiety disorders are the most common mental health condition(1), with a lifetime prevalence of 28.8%(2) and a median age of onset of 11 years(2). As such, they are the most common emotional disorders in childhood(3, 4) , with worldwide prevalence rates of 6.5% in children and adolescents(5) and are associated with an increased risk of subsequent mental health problems, substance abuse and poor educational attainment(6, 7). This level of impairment results in a high economic and societal burden(8), with anxiety disorders being the leading cause of years lived with disability (YLDs)(9). As such, there is a clear need for early identification and access to evidence-based interventions(10). Effective treatments for childhood anxiety disorders exist(11, 12), however, only a minority of affected children access this support, with lower rates of treatment access than externalizing disorders (such as ADHD or Conduct Disorder) and mood disorders(13, 14).

In the UK, two-thirds of children see their General Practitioner (GP) at least once a year(15) and there has been a steady rise in the number of children and young people presenting in primary care with mental health difficulties(16, 17). GPs are often the first medical professionals that families see when they have mental health concerns and are in a position to develop strong relationships with families, in a non-stigmatising setting(18) and are as often seen as "gate-keepers" to accessing mental health services(19, 20). However, many GPs report a lack of confidence in their competence and skills in child and adolescent mental health, feel that they need further training, (21), and believe that their role in this area requires further research and definition(22). In addition, they face barriers such as insufficient resources when managing mental health conditions in primary care and extensive waiting lists for specialist services(23) and a high rate of rejected referrals. The reason for this is unclear, possibly reflecting issues with GP detection of mental health problems in this age group and lack of clarity regarding thresholds for CAMHS amongst GPs, associated with a lack decision making aids for child and adolescent mental health disorders**.**(24).

The identification and management of childhood anxiety disorders may present a particular challenge for GPs due to their broad clinical presentation, frequent co-morbidity with other mental health problems(2) and the common reliance on parents/caregivers to seek help(25). Indeed, anxiety disorders are known to be less likely to be recognized by primary health care professionals than other childhood mental health problems(26), potentially due to anxiety disorders being seen as less burdensome for the parents than externalizing disorders (27). In a recent qualitative study, there was wide variation in GPs' confidence managing anxiety disorders in (pre-adolescent) children, with many GPs describing feeling ill-equipped to manage and support children with these disorders(28). While many of the barriers they described were similar to those experienced by GPs in relation to other mental health problems in children (e.g. time constraints, difficulties accessing specialist help), GPs highlighted particular difficulties distinguishing anxiety disorders from physical conditions. It was also notable that they attributed their confidence in managing childhood anxiety disorders not only to factors such as training background and practice characteristics but also to whether they were a parent themselves.



For peer review only - http://bmjopen.bmj.com/site/about/guidelines.xhtml

Given the substantial burden that anxiety disorders in pose in childhood, and beyond, successful early identification and appropriate management is imperative. However, as previous research has shown, GPs face numerous internal and external barriers in dealing with mental health disorders, with some early indication that anxiety disorders may bring specific challenges. This research aims to build on the findings of the previous qualitative work(28) to ascertain a more representative and generalisable view of these issues throughout the country. In addition to this, the study aims to develop understanding of factors that influence GPs confidence in dealing with these disorders.

*Research questions*

RQ1: i) What are the barriers and facilitators to (a) identification, (b) management and (c) referral to specialist services for pre-adolescent children with anxiety disorders for General Practitioners in England?

RQ2 ii) What practice and personal factors are associated with GPs' confidence in identifying and managing childhood anxiety disorders?



For peer review only - http://bmjopen.bmj.com/site/about/guidelines.xhtml

# Methods

## Procedure

Ethical approval for this study was granted by the School of Psychology & Language Science Ethics Board at the University of Reading. Approval was also granted by the Health Research Authority to conduct the study with NHS staff.

Participants completed a 50 item self-report online questionnaire (approx. 15 mins) which contained three sections, addressing a) identification, b) management and c) access to specialist services for children under 12 years with a suspected anxiety disorder (see Appendix 1). Free-text boxes for additional barriers were included at the of each section, and at the end of the survey for any further comment. The content of the survey was informed by findings from a preceding systematic review(23) and qualitative study(28) which highlighted the utility of separating the stages of the primary care process. The questions were developed through an iterative process incorporating feedback from an academic General Practitioner (GP) and the research team.

The first half of each section contained questions about the GP's confidence and beliefs (e.g. "I feel confident identifying anxiety disorders in children under 12 years"). Based on the previous qualitative research, endorsement of these items indicated facilitators (and is labelled as such in the results section). The second half of each section asked GPs to rate external barriers they faced (e.g. "language barriers"). Responses to all items were rated on a 5 point Likert scale (e.g. from 'completely disagree' to 'completely agree', or "not at all" to "very much").

GPs provided information on gender, age, number of years qualified, whether they had completed psychiatric or paediatric training, whether were a parent (including child's age), and whether they considered themselves 'research active'. Information on their practice population, such as size, socio-economic-status and ethnic diversity was obtained via their practice postcode.

## Participants

*A priori* sample size estimates (https://www.qualtrics.com/blog/calculating-sample-size/) indicated that at least 381 General Practitioners were required to estimate the frequency of particular barriers with 95% confidence intervals, based on a total population of approx. 41,985 GPs in England(29), and to allow for robust analyses using a logistic regression with 10 predictor variables(30).

Participants were GPs currently working in the National Health Service. There were no exclusion criteria. GPs were recruited through the National Institute for Health Research (NIHR) Clinical Research Networks (CRNs). Recruitment methods varied by CRN, from mass emails to all registered GPs in the region, to more targeted recruitment, including talks at GP forums. Multiple GPs per practice were allowed. Recruitment took place from the 15$^{th}$ July 2016 – 6$^{th}$ Feb 2017 and covered 11 CRNS (out of a possible 15). CRNs contacted GPs


For peer review only - http://bmjopen.bmj.com/site/about/guidelines.xhtml

within their regions via an email which contained a link to the survey. Paper copies were available on request.

Participation in this study was anonymous, although GPs' surgery postcode was requested to retrieve demographic information about the practice, and for CRN recruitment purposes. All participants were required to give informed consent.

**Patient and public involvement**

No patients were involved in this research. One academic GP was involved in piloting the questionnaire.

**Statistical Methods**

Statistical analyses were performed using IBM SPSS(V22)(31). As there was little missing data, cases which had missing data were excluded from analysis (number of cases missing per variable ranged between 7-20). To address research question 1, frequencies, including confidence intervals, are reported for items on the questionnaire. To address research question 2, logistic regression analyses were conducted with the dependent variables of confidence a) identifying[2] and b) managing[2] anxiety disorders in children under 12 years. These dependent variables were derived from the questions: 'I am confident identifying anxiety disorders' and 'I am confident managing anxiety disorders'. These questions were transformed into a binary format by collapsing; 'agree' and 'completely agree' together and 'neutral', 'disagree' and 'completely disagree' together. The following predictors were included: patient population size[1], deprivation decile of practice (0-10, with 10 being the least deprived)[1], ethnicity of patient population (% non- BAME)[1], psychiatric/ paediatric rotation completed as part of GP training[2], gender[2], number of years since qualification as a GP[2] and whether they were a parent of a child over the age of 5 years[2] (i.e. have personal experience of parenting a school-aged child). These factors were chosen based on previous qualitative research which indicated the potential influence they have on a GP's confidence.

The free-text comments were content-coded and organised into broad categories.

1 Available on Public Health England

2 Self-report



For peer review only - http://bmjopen.bmj.com/site/about/guidelines.xhtml

## Results

A total of 971 GPs completed the questionnaire. See Table 1 for demographic information for a breakdown of participant numbers by Clinical Research Networks.

Table 1. Characteristics of GPs and their practices.

| **Variable** | | **Missing** |
|---|---|---|
| *GP* | | |
| % female | 52.70% | 9 |
| Number of years qualified | M = 14.9 (Range: <1 - 43) | 7 |
| Psychiatric rotation | 47.30% | 16 |
| Paediatric rotation | 73.50% | 11 |
| Child over 5 years old | 72% | 9 |
| Referred a child to specialist services for an anxiety disorder more than 5 times in last 5 years | 58.8 | |
| *Practice* | | |
| Number of registered patients | M = 12,009 (Range: 1503 - 55499) | 19 |
| SES of patients (deprivation decile) | M = 6.7 (Range: 1-10) | 19 |
| % non-white ethnic groups | M = 7% (Range: .8 - 72.2) | 20 |

i) What are the barriers and facilitators to the identification, management and referral of children, under the age of 12 years, with anxiety disorders for General Practitioners in the UK?

Table 2. (below) reports the facilitators and barriers to identification, management and referral, as reported by GPs. A composite of those who agreed and strongly agreed, as well as those who disagreed and strongly disagreed, is presented for each item, in addition to the 95% confidence interval.

The table demonstrates that although GPs feel that it is their responsibility and feel comfortable discussing anxiety disorders with families, inadequate training, as well as time restrictions and a lack of accessible treatment pose the biggest barriers for GPs in identification of childhood anxiety disorders. A lack of confidence, training, resources and time were the biggest barriers to management. GPs believed that specialist services were effective but waiting times posed the largest barrier to access and less than 10% of GPs felt that they had a relationship with these services. Stigma, language and culture issues were not seen as barriers to GPs' identification, management of referral.

For peer review only

Table 2. GP endorsement of facilitators and barriers to the identification, management and referral of childhood anxiety disorders

| **Item** | **% "agree" + "completely agree"** | **95% confidence interval** | | **% "disagree" + "completely disagree"** | **95% confidence interval** | |
|---|---|---|---|---|---|---|
| ***Identification of anxiety disorders in children under 12 years*** | | *Lower* | *Upper* | | *Lower* | *Upper* |
| *Facilitators* | | | | | | |
| Feel confident in ability | 53 | 50.78 | 57.06 | 12 | 9.91 | 13.99 |
| Believe training was adequate | 21 | 19.09 | 24.29 | 48 | 44.68 | 50.98 |
| Comfortable broaching idea of anxiety disorders with the child | 80 | 77.72 | 82.74 | 7 | 5.14 | 8.30 |
| Comfortable broaching idea of anxiety disorders with the family | 88 | 86.81 | 90.79 | 3 | 1.92 | 4.08 |
| Believe it is part of a GP's responsibility | 93 | 91.60 | 94.78 | 1 | 0.54 | 1.93 |
| *Endorsement of barriers* | **% "quite a lot" + "very much"** | | | **% "a little" + "not at all"** | | |
| Limitations in children's communication abilities | 21 | 19.05 | 24.24 | 33 | 29.93 | 35.84 |
| Misinformation from parents | 27 | 24.06 | 29.65 | 35 | 32.52 | 38.55 |
| Concerns about stigmatising the child | 10 | 8.68 | 12.56 | 69 | 66.77 | 72.56 |
| Family concerns about stigma | 16 | 13.60 | 18.21 | 60 | 57.25 | 63.41 |
| Cultural barriers | 17 | 14.46 | 19.17 | 58 | 54.89 | 61.11 |
| Language barriers | 18 | 15.83 | 20.69 | 64 | 61.38 | 67.41 |
| Family reluctance to accept | 20 | 17.73 | 22.80 | 43 | 39.80 | 46.03 |

For peer review only

| | | | | | | |
|---|---|---|---|---|---|---|
| disorder | | | | | | |
| Time restrictions | 67 | 63.91 | 69.84 | 14 | 12.33 | 1.677 |
| Lack of available/accessible treatment | 68 | 98.34 | 99.61 | 15 | 12.91 | 17.42 |
| Lack of effective treatment | 45 | 42.53 | 48.80 | 27 | 23.91 | 29.48 |
| ***Management of children under 12 years with anxiety disorders*** | **% "agree" + "completely agree"** | | | **% "disagree" + "completely disagree"** | | |
| *Facilitators* | | | | | | |
| Feel confident in ability | 13 | 11.32 | 15.64 | 51 | 48.71 | 55.02 |
| Believe training was adequate | 10 | 8.29 | 12.09 | 66 | 63.56 | 69.50 |
| Comfortable discussing management strategies with the family | 52 | 49.33 | 55.63 | 20 | 17.32 | 22.34 |
| Comfortable discussing management strategies with the child | 46 | 43.29 | 49.58 | 22 | 19.21 | 24.42 |
| Aware of resources to aid families | 24 | 21.54 | 26.95 | 75 | 73.05 | 78.46 |
| Believe having a relationship with a family is beneficial | 94 | 92.54 | 95.52 | <1 | 0.39 | 1.66 |
| Aware of local agencies to support children and their families | 65 | 63.06 | 69.03 | 17 | 15.09 | 19.89 |
| Believe it is part of a GP's role | 69 | 66.70 | 72.49 | 10 | 8.01 | 11.77 |
| *Endorsement of barriers* | **% "quite a lot" + "very much"** | | | **% "a little" + "not at all"** | | |
| Cultural barriers | 14 | 12.01 | 16.43 | 60 | 58.72 | 64.86 |

For peer review only

| | | | | | | |
|---|---|---|---|---|---|---|
| Language barriers | 16 | 13.98 | 18.65 | 64 | 61.32 | 67.37 |
| Time restrictions | 72 | 70.15 | 75.76 | 11 | 9.68 | 13.73 |
| Family reluctance to accept the disorder | 19 | 16.78 | 21.76 | 46 | 43.38 | 49.68 |
| Limitations in children's communication abilities | 18 | 15.80 | 20.67 | 43 | 40.60 | 46.86 |
| Misinformation from parents | 18 | 15.55 | 20.40 | 50 | 47/78 | 54.09 |
| Concerns about stigmatising the child | 7 | 5.91 | 9.25 | 71 | 69.31 | 74.98 |
| Family concerns about stigma | 11 | 9.23 | 13.21 | 63 | 60.58 | 66.66 |
| ***Referral of children under 12 years with anxiety disorders*** | **% “agree” + “completely agree”** | | | **% “disagree” + “completely disagree”** | | |
| *Facilitators* | | | | | | |
| Long waiting times reduce the likelihood of making a referral | 50 | 47.37 | 53.66 | 35 | 32.65 | 38.68 |
| Parental pressure increases the likelihood of making a referral | 85 | 83.18 | 87.62 | 5 | 4.02 | 6.87 |
| Have a relationship with local specialist services | 6 | 4.30 | 7.24 | 79 | 76.49 | 81.62 |
| Believe that specialist services' interventions are effective | 78 | 75.63 | 80.83 | 4 | 3.22 | 5.84 |
| *Endorsement of barriers* | **% “quite a lot” + “very much”** | | | **% “a little” + “not at all”** | | |
| Long waiting times | 89 | 87.03 | 90.97 | 3 | 1.91 | 4.04 |
| Cultural barriers | 8 | 6.22 | 9.65 | 76 | 75.11 | 80.38 |
| Language barriers | 8 | 6.83 | 10.40 | 77 | 76.49 | 81.66 |
| Time restrictions | 30 | 28.26 | 34.14 | 51 | 49.40 | 55.73 |

For peer review only



For peer review only - http://bmjopen.bmj.com/site/about/guidelines.xhtml

| | | | | | | |
|---|---|---|---|---|---|---|
| Family reluctance to accept the disorder | 12 | 10.47 | 14.68 | 62 | 60.68 | 66.78 |
| Limitations in children's communication abilities | 6 | 4.91 | 8.03 | 70 | 68.19 | 73.93 |
| Misinformation from parents | 9 | 7.83 | 11.59 | 66 | 64.11 | 70.06 |
| Concerns about stigmatising the child | 6 | 4.55 | 7.57 | 78 | 76.94 | 82.06 |
| Family concerns about stigma | 8 | 6.71 | 10.24 | 73 | 71.80 | 77.32 |
| Lack of available/accessible treatment | 54 | 51.78 | 58.10 | 28 | 25.49 | 31.22 |

For peer review only



For peer review only - http://bmjopen.bmj.com/site/about/guidelines.xhtml

ii) What factors are associated with a GPs confidence identifying managing this disorder?

A: The regression model (see Table 3) was a significant fit overall ($\chi^2$ (1) = 16.7, p < .05). Being female was significantly associated with higher confidence identifying anxiety disorders in children, (Ex(B) = 1.4, 95%CI 1.1-1.9), as was being in a less deprived practice (Ex(B) = 1.1), 95%CI 1-1.1. GP psychiatric and paediatric training, as well as years of experience and parenthood did not have any impact on their confidence, nor did size or ethnicity of their practice population.

Table 3. Regression model of factors are associated with a GP's confidence identifying childhood anxiety disorders

| | B | Exp(B) | 95% C.I. for EXP(B) | |
|---|---|---|---|---|
| | | | Lower | Upper |
| *GP* | | | | |
| Gender | .352* | 1.423 | 1.090 | 1.856 |
| Psychiatry training | .147 | 1.158 | .889 | 1.510 |
| Paediatric training | .155 | 1.168 | .861 | 1.5834 |
| Years qualified | -.004 | .996 | .980 | 1.012 |
| Child over 5 years | .163 | 1.177 | .841 | 1.656 |
| *Practice* | | | | |
| No. of patients | .000 | 1.000 | 1.000 | 1.000 |
| SES (deprivation decile) | .062* | 1.064 | 1.006 | 1.125 |
| % non-white ethnic groups | .008 | 1.008 | .995 | 1.021 |
| Constant | .081 | .922 | | |

* Note. $R^2$ =. 01 (Hosmer & Lemeshow) .01 (Cox & Snell) .03 (Nagelkerke). Model $\chi$2( 1) = 17.4, p < .05.

(ii) What factors are associated with a GPs confidence managing this disorder?

B: The regression model (see Table 4) was significant overall ($\chi^2$(1) = 18.2, p < .05.). Having a child over the age of 5 was significantly associated with greater confidence managing anxiety disorders in children (Ex(B)=2), 95%CI 1.1-3.5, as was being in a less deprived practice (Ex(B)=1.1, 95%CI 1-1.2). GP psychiatric and paediatric training, as well as years of experience and gender did not have any impact on their confidence, nor did size or ethnicity of their practice population.

Table 4. Regression model of factors which are associated with a GP's confidence managing childhood anxiety disorders

| | B | Exp(B) | 95% C.I. for EXP(B) | |
|---|---|---|---|---|
| | | | Lower | Upper |
| *GP* | | | | |
| Gender | -.239 | .787 | .531 | 1.168 |
| Psychiatry training | .273 | 1.314 | .887 | 1.947 |

For peer review only



For peer review only - http://bmjopen.bmj.com/site/about/guidelines.xhtml

| | | | | |
|---|---|---|---|---|
| Paediatric training | .218 | 1.244 | .766 | 2.019 |
| Years qualified | .002 | 1.002 | .979 | 1.027 |
| Child over 5 years | .687* | 1.987 | 1.132 | 3.489 |
| *Practice* | | | | |
| No. of patients | .000 | 1.000 | 1.000 | 1.000 |
| SES (deprivation decile) | .090* | 1.094 | 1.004 | 1.193 |
| % non-white ethnic groups | .015 | 1.015 | .997 | 1.034 |
| Constant | -1.847 | .158 | | |

* Note. $R^2$ =. 05 (Hosmer & Lemeshow) .02 (Cox & Snell) .04 (Nagelkerke). Model $\chi2( 1) = 20.23$, $p = .01$.

For peer review only



For peer review only - http://bmjopen.bmj.com/site/about/guidelines.xhtml

## Discussion

*Summary*

Although most GPs believe that it is their responsibility to identify and manage anxiety disorders in children, only half of those surveyed felt confident identifying, and only 13% felt confident managing these disorders. These figures may not be surprising given that only 21% of GPs believed their training in identification was adequate, just 10% believed that their training in management of anxiety disorders was adequate, and the vast majority were not aware of any resources to provide families. Time restrictions also posed a barrier for both identification and management. Although 78% of GPs believed that specialist services' interventions were likely to be effective, long waiting times and lack of accessible treatments were overwhelmingly endorsed as a barrier to accessing specialist services and 79% felt that they did not have a relationship with such services. Free-text comments for this section highlighted that GPs felt CAMHS referral criteria were unclear, and often had very high thresholds. However, notably 85% indicated that referral was significantly increased by parental pressure, demonstrating the need for parents to be strong advocates for their children in order to access specialist services. Interestingly, although previous literature reported stigma surrounding mental health as a barrier for GPs, this was not the case for the GPs in this research.

Female GPs and those in less deprived practices were associated with more confidence identifying child anxiety disorders. Notably, GPs who had completed psychiatric or paediatric training were not associated with more confidence in identification, reinforcing the commonly reported view that the training received was inadequate. In terms of management, GPs in less deprived practices and those who were parents of a child over the age of five were associated with more confidence. This latter finding was particularly striking and was consistent with our previous qualitative work that suggested that GPs rely on their own personal experience, as their professional training was largely viewed as inadequate, to manage these disorders(28).

*Strengths and limitations*

One of the key strengths of the study is the inclusion of a large, nationally representative group of GPs. Another is that it used findings from a recent systematic review(23), qualitative study(28) and GP feedback to inform the development of the questionnaire, to ensure that the questions were meaningful to the GPs and that the most pertinent questions were asked. This appeared to have been successful as the free-text responses mainly reinforced the findings from the main questionnaire and little new information was added. Unlike many previous studies, the questionnaire distinguished between different stages of the primary care process (i.e. identification, management, referral to specialist services). This allows pinpointing of where issues are most problematic, for example, time restrictions were a barrier for identification and management, but much less so for referral. We can also see that GPs feel significantly more confident identifying anxiety disorders than managing them. Future studies would benefit from examining these stages at a more granular level, for



For peer review only - http://bmjopen.bmj.com/site/about/guidelines.xhtml

example, to further understand the skills and confidence necessary at different stages of the process e.g. identifying the problem, broaching the subject with the family/young person.

These strengths need to be considered in the light of various limitations. Efforts were made to recruit a number of GPs to pilot the survey, however only one responded, which is a limitation of the study. Although the sample had similar demographic characteristics to GPs across England and we recruited a substantial proportion of GPs (n= 443; 46%) who did not consider themselves 'research active', no data were available on those who chose not to participate and we cannot exclude the possibility of a self-selection bias influencing the findings. We also did not assess GPs' abilities to accurately identify anxiety disorders in children and cannot rule out the possibility that in some cases GPs answered questions in relation to mental health disorders other than anxiety. This survey involved GPs reporting on their own experiences, which may be influenced by poor recall and social desirability biases. Furthermore, as this survey is cross-sectional, conclusions cannot be drawn about causality. Most free text comments related to questions asked in the survey, however an additional area that GPs raised in the comments was the role of others in identification of anxiety disorders and for referrals to specialist services, in particular, schools and health visitors. A final limitation of this study is the lack of distinction between anxiety disorders, which are not a homogenous group, as well as the fact that we focused on anxiety disorders as a distinct condition, despite the fact that they are often co-morbid with other psychological and physical health disorders. This may limit the generalisability of this study.

*Comparison with previous research*

The specific focus on anxiety disorders is a further strength of this research given the specific challenges faced with this condition, and this contrasts with much of the previous work in this area which does not distinguish between mental health disorders. Many of the findings in this study echo previous qualitative findings(24, 28), such as the positive impact of being a parent on confidence managing child anxiety disorders and issues associated with long waiting times and poor access to specialist services. It also reinforced the idea that GPs working in more deprived areas may experience particular difficulties potentially due to more complex family situations. Future studies would benefit from a more in-depth examination into the association between deprivation and GP confidence in identification and management. However, although some of the GPs interviewed in the previous research seemed to have some uncertainty regarding their role in the identification and management of this condition, it appeared that the majority of GPs surveyed believed that this is their responsibility, at least in part. This research also reinforced a recent stem4 report which found that GPs had serious concerns about accessing specialist help for young people with mental health disorders(32). The issue of poor access to services and long waiting times are common across the mental health literature, but this study highlights a number of novel findings, such as the widespread lack of confidence in management of childhood anxiety disorders, and the stronger role of personal experience of being a parent than access to relevant specialist training in GP confidence managing childhood anxiety disorders.

*Implications for clinicians, policy-makers and researchers*



For peer review only - http://bmjopen.bmj.com/site/about/guidelines.xhtml

The focus of this research was to investigate barriers, from a GP's perspective, however in order to fully understand how to increase access to appropriate support for childhood anxiety disorders, future studies could seek to capture objective data (e.g. referral data), in order to corroborate the questionnaire findings. This would be useful to understand whether self-report correlates with actual behavior. Furthermore, providing GPs with vignettes describing children with and without anxiety disorders may be useful to determine whether a GP's perception of their confidence is related to their actual identification abilities.

This research strongly suggests that GPs' current training in childhood anxiety disorders is inadequate, and highlights the need to place increased emphasis on training, both in medical school training and beyond, to boost confidence and develop skills. This reinforces previous qualitative findings which highlight that GPs believed that their training in mental health problems had been inadequate, and focused on adults to the detriment of children and young people(33). This training should be meaningful for GPs and firmly grounded in the context of primary care, as well as focused specifically on child and adolescent populations. This supports recommendations from the RCGP that GPs should receive specialist-led training in child health and mental health problems(34). It is encouraging that the majority of GPs surveyed see identifying and managing this condition as part of their role, in addition to other organisations (e.g. schools), but it is possible that a lack of confidence and comfort with the topic matter prevents GPs from fully engaging with a condition(35). In addition to further training, previous qualitative research suggests that increased contact and collaboration with specialist services would increase GPs confidence in this area(33). Furthermore, as with previous studies in this area, difficulty accessing specialist services in a timely manner poses a significant barrier for GPs who commonly used the free-text comments to express the view that CAMHS' referral thresholds are prohibitively high, and service inclusion criteria are unclear. Increasing GPs confidence in this area, as well as providing them with more effective resources, may encourage and enable GPs to manage many cases in primary care and only refer severe or complex cases, thus alleviating pressure on specialist services.

**Conclusion**

Failing to intervene effectively with childhood anxiety disorders brings negative implications for both individual children and families(6) and increased costs for society(8). GPs consider identification and management of childhood anxiety disorders to be within their role however they face a number of barriers and lack confidence in their abilities. Increased GP training in identification and management of child anxiety disorders may help to strengthen GPs' confidence and ability to effectively support children under the age of 12 with anxiety disorders. Furthermore, the findings suggest that GPs would benefit from increased access to ongoing training and appropriate resources to support identification and early management and to facilitate better communication around access to specialist services for children with anxiety disorders.



For peer review only - http://bmjopen.bmj.com/site/about/guidelines.xhtml

**Acknowledgements:** We acknowledges the support of the following National Institute for Health Research Clinical Research Networks (NIHR CRN): Thames Valley & South Midlands, East Midlands, Eastern, North West London, South London, North East and North Cumbria, South West Peninsula, West Midlands, Yorkshire & Humber, North Thames, Wessex.

For peer review only



For peer review only - http://bmjopen.bmj.com/site/about/guidelines.xhtml

# References


1. Kessler RC, Ruscio AM, Shear K, Wittchen H-U. Epidemiology of anxiety disorders. Behavioral neurobiology of anxiety and its treatment: Springer; 2009. p. 21-35.
2. Kessler R, C.;, Berglund P, Demler O, Jin R, Merikangas KR, Walters EE. Lifetime Prevalence and Age-of-Onset Distributions of DSM-IV Disorders in the National Comorbidity Survey Replication. JAMA Psychiatry. 2005;62(6):593.
3. Lawrence D, Hafekost J, Johnson SE, Saw S, Buckingham WJ, Sawyer MG, et al. Key findings from the second Australian Child and Adolescent Survey of Mental Health and Wellbeing. Aust N Z J Psychiatry. 2016;50(9):876-86.
4. Merikangas KR, He JP, Burstein M, Swanson SA, Avenevoli S, Cui L, et al. Lifetime prevalence of mental disorders in U.S. adolescents: results from the National Comorbidity Survey Replication--Adolescent Supplement (NCS-A). J Am Acad Child Adolesc Psychiatry. 2010;49(10):980-9.
5. Polanczyk GV, Salum GA, Sugaya LS, Caye A, Rohde LA. Annual Research Review: A meta-analysis of the worldwide prevalence of mental disorders in children and adolescents. J Child Psychol Psychiatry. 2015;56(3):345-65.
6. Woodward LJ, Fergusson DM. Life course outcomes of young people with anxiety disorders in adolescence. J Am Acad Child Adolesc Psychiatry. 2001;40(9):1086-93.
7. Knapp M, Snell T, Healey A, Guglani S, Evans-Lacko S, Fernandez JL, et al. How do child and adolescent mental health problems influence public sector costs? Interindividual variations in a nationally representative British sample. J Child Psychol Psychiatry. 2015;56(6):667-76.
8. Fineberg NA, Haddad PM, Carpenter L, Gannon B, Sharpe R, Young AH, et al. The size, burden and cost of disorders of the brain in the UK. J Psychopharmacol. 2013;27(9):761-70.
9. Kassebaum NJ, Arora M, Barber RM, Bhutta ZA, Brown J, Carter A, et al. Global, regional, and national disability-adjusted life-years (DALYs) for 315 diseases and injuries and healthy life expectancy (HALE), 1990-2015: a systematic analysis for the Global Burden of Disease Study 2015. The Lancet. 2016;388(10053):1603.
10. Craske MG, Stein MB. Anxiety. The Lancet. 2016;388(10063):3 048-4059.
11. James AC, James G, Cowdrey FA, Soler A, Choke A. Cognitive behavioural therapy for anxiety disorders in children and adolescents. Cochrane Database Syst Rev. 2013(6):CD004690.
12. Zhen W, H. WSP, Leslie S, Wigdan F, S. MA, Mouaz A, et al. Comparative Effectiveness and Safety of Cognitive Behavioral Therapy and Pharmacotherapy for Childhood Anxiety Disorders A Systematic Review and Meta-analysis. JAMA Paediatrics. 2017.
13. Merikangas KR, He JP, Burstein M, Swendsen J, Avenevoli S, Case B, et al. Service utilization for lifetime mental disorders in U.S. adolescents: results of the National Comorbidity Survey-Adolescent Supplement (NCS-A). J Am Acad Child Adolesc Psychiatry. 2011;50(1):32-45.
14. Essau CA. Frequency and patterns of mental health services utilization among adolescents with anxiety and depressive disorders. Depress Anxiety. 2005;22(3):130-7.
15. Hippisley-Cox, J, Y. V. Trends in consultation rates in general practice 1995/1996 to 2008/2009: analysis of the QResearch database. . . 2009.




For peer review only - http://bmjopen.bmj.com/site/about/guidelines.xhtml

16. Charles J, Harrison CM, Britt H. Management of children's psychological problems in general practice 1970-1971, 1990-1991 and 2008-2009. Australian & New Zealand Journal of Psychiatry. 2011;45(11):976-84.
17. Kelleher KJ, McInerny TK, Gardner WP, Childs GE, Wasserman RC. Increasing identification of psychosocial problems: 1979-1996. Pediatrics. 2000;105(6):1313-21.
18. Roberts JH, Bernard PM. 'Can he have the test for bipolar, doctor? His dad's got it': Exploring the potential of general practitioners to work with children and young people presenting in primary care with common mental health problems - A clinical initiative. Mental Health in Family Medicine. 2012;9(2):115-23.
19. Committee oH. The future of pediatrics: Mental health competencies for pediatric primary care. Pediatrics. 2009;124(1):410-21.
20. Department for Children SaF. *Early intervention: securing good outcomes for all children and young people*Early intervention: securing good outcomes for all children and young people. London: *Department for Children, Schools and Families*Department for Children, Schools and Families; 2010.
21. Roberts JH, Crosland A, Fulton J. "I think this is maybe our Achilles heel..." exploring GPs' responses to young people presenting with emotional distress in general practice: a qualitative study. BMJ Open. 2013;3(9):e002927.
22. Chien A, Coker T, Choi L, Slora E, Bodnar P, Weiley V, et al. What do pediatric primary care providers think are important research questions? A perspective from PROS providers. Ambul Pediatr. 2006;6(6):352-5.
23. O'Brien D, Harvey K, Howse J, Reardon T, Creswell C. Barriers to managing child and adolescent mental health problems: a systematic review of primary care practitioners' perceptions. Br J Gen Pract. 2016:bjgpoct-2016-66-651-harvey-fl-p.
24. Hinrichs S, Owens M, Dunn V, Goodyer I. General practitioner experience and perception of Child and Adolescent Mental Health Services (CAMHS) care pathways: a multimethod research study. BMJ Open. 2012;2(6).
25. Sayal K, Taylor E. Detection of child mental health disorders by general practitioners. British Journal of General Practice. 2004;54(502):348-52.
26. Burke DA, Koot HM, de Wilde A, Begeer S. Influence of Child Factors on Health-Care Professionals' Recognition of Common Childhood Mental-Health Problems. Journal of child and family studies. 2016;25(10):3083-96.
27. Jacobs CO, Loades ME. An investigation into GPs' perceptions of children's mental health problems. Child and Adolescent Mental Health. 2016;21(2):90-5.
28. O'Brien D, Harvey K, Reardon T, Young B, Creswell C. General Practitioners' Experiences of Children with Anxiety
Disorders in Primary Care: a Qualitative Study. British Journal of General Practice. 2017.
29. Digital GWTN. General and Personal Medical Services,
England September 2015 - March 2016. Health and Social Care Information Centre; 2016.
30. Peduzzi P, Concato J, Kemper E, Holford TR, Feinstein AR. A simulation study of the number of events per variable in logistic regression analysis. Journal of clinical epidemiology. 1996;49(12):1373-9.
31. Corp I. IBM SPSS Statistics for Windows, Version 22.0. Armonk, NY: IBM Corp; 2013.
32. stem4. "A Time Bomb Waiting to Explode" A Survey of GPs' Concerns about Mental Health Services for Children and Young People. London; 2016.
33. O'Brien D, Harvey K, Reardon T, Young B, Creswell C. General Practitioners' Experiences of Children with Anxiety

For peer review only



For peer review only - http://bmjopen.bmj.com/site/about/guidelines.xhtml

Disorders in Primary Care: a Qualitative Study. British Journal of General Practice. 2017.
34. Practice RCoG. Enhanced and extended GP training progress update. London; 2013.
35. Salmon P, Peters S, Clifford R, Iredale W, Gask L, Rogers A, et al. Why do general practitioners decline training to improve management of medically unexplained symptoms? Journal of General Internal Medicine. 2007;22(5):565-71.

For peer review only



For peer review only - http://bmjopen.bmj.com/site/about/guidelines.xhtml

**Author Statement:** All three authors (KH, DOB, CC) were involved in the design of this study. Participant recruitment and analysis was conducted by DOB. The main draft of the manuscript was written by DOB with input by KH and CC.

**Data sharing statement:** Full dataset and statistical codes are available from the corresponding author. Data supporting the results reported in this paper are openly available from the University of Reading Research Data Archive at http://dx.doi.org/10.17864/1947.192

**Acknowledgements**

The authors would like to thank the National Institute for Health Research Clinical Research Networks for all of their help and support with recruitment for this study. They would also like to thank all of the General Practitioners who participated in this research.

For peer review only



For peer review only - http://bmjopen.bmj.com/site/about/guidelines.xhtml

527



For peer review only - http://bmjopen.bmj.com/site/about/guidelines.xhtml

# CADPC
## Consent page

### Children with Anxiety Disorders in Primary Care: the views of General Practitioners II

We invite you to take part in a National Institute of Health Research funded study which focuses on management of childhood anxiety disorders (children under 12 years) by General Practitioners in England. This study explores GPs' experiences of identification, management and referral of these disorders.

**Please note, while some of these questions may seem repetitive, consider whether they are being asked in relation to either identification, management or referral.** Your responses will help inform practice guidelines for children with anxiety disorders.

**Taking part in this study will involve completing a survey, which should take 10-15 minutes to complete. This is open to as many GPs per surgery as desired.**

As a reimbursement for taking part in the study you (or a nominated member of your practice) will have the opportunity to take part in a Webinar about identification and management of childhood anxiety disorders. This module may be used to contribute towards Continued Professional Development points and a certificate of attendance outlining your involvement in important mental health research will be provided. You will also be entered into a prize draw to win one of two £100 Amazon vouchers.

We are happy to answer queries about the study, please contact the researchers for further information:

Doireann O'Brien, B.A., M.Sc.
Email: doireann.obrien@pgr.reading.ac.uk

Professor Cathy Creswell
Tel: +44 (0)118 378 6798

Further details of the study are available as an information sheet. Please find information sheet enclosed.

**All items of the consent form, below, must be initialled to indicate consent.**

For peer review only

I can confirm that I have read and understand the information sheet, have had the opportunity to ask questions and, if applicable, have had these questions answered satisfactorily ⓘ *Required* ☐

I understand that my participation is voluntary, that I can withdraw my consent at any time, without giving a reason and without my professional role being compromised ⓘ *Required* ☐

I understand that the answers to my questions will be stored in a secure location, but will bear no identifying information. I also understand that the data will be kept for 12 months and after this time they will be destroyed ⓘ *Required* ☐

I understand that, should I lose the capacity to consent to be in the study, the research centre will retain any information collected prior to this point ⓘ *Required* ☐

I understand that relevant sections of my data collected during the study, may be looked at by individuals from the University of Reading, from regulatory authorities or from the NHS Trust, where it is relevant to my taking part in this research. I give permission for these individuals to have access to my records ⓘ *Required* ☐

I agree to take part in the study ⓘ *Required* ☐

For peer review only

# SURVEY

| | |
|---|---|
| Age: *(please circle)* 23-35 35-45 45-55 55-65 65+ | |
| Identify my gender as: *(please circle)* MALE FEMALE OTHER | |
| Psychiatric rotation completed as part of GP training: *(please circle)* YES NO | |
| Paediatric rotation completed as part of GP training: *(please circle)* YES NO | |
| Year qualified as a GP: | |
| Practice Name: | |
| Practice Postcode: | |
| Years employed in current Trust: | |
| Would you consider yourself research active? *(please circle)* YES NO | |
| Are you a parent or guardian? *(please circle)* YES NO | |
| If so, what ages are your children: | |
| Compared to other GPs in your practice, do you see more or fewer child patients? *(please circle)* YES NO SIMILAR AMOUNT | |

For peer review only

PLEASE NOTE: **ALL QUESTIONS ARE IN RELATION TO ANXIETY IN CHILDREN AGED 12 & UNDER (i.e. primary school age and below)**

For each item identified below, circle the number to the right that best fits your judgment of its quality.
Use the rating scale to select the number.

| **Survey Item** | **Scale***<br>**1 = completely disagree**<br>**2 = disagree**<br>**3 = neutral**<br>**5 = agree**<br>**5 = completely agree**<br><br>*unless otherwise specified | | | | |
|---|---|---|---|---|---|
| **Identification**<br>*These questions are in relation to initially identifying the presence of an Anxiety Disorder in a child* | | | | | |
| 1. How common are anxiety disorders in children in your experience? | 1 in 3 | 1 in 10 | 1 in 15 | 1 in 20 | 1 in 40 |
| 2. How often do you see anxiety disorders in your practice? | | | | | |
| 3. I believe that anxiety disorders are prevalent in children under 12 | 1 | 2 | 3 | 4 | 5 |
| 4. I am confident in my ability to recognise anxiety disorders in children | 1 | 2 | 3 | 4 | 5 |
| 5. I am regularly looking out for anxiety disorders in children | 1 | 2 | 3 | 4 | 5 |
| 6. My training adequately equipped me with the skills to identify anxiety disorders in children | 1 | 2 | 3 | 4 | 5 |
| 7. I use screening tools to aid identification of anxiety disorders in children (Screening tools refer to validated measures used to aid identification of a suspected anxiety disorder) | Yes | | | No | |
| a. *If yes:* I find screening tools useful for the identification of anxiety disorders in children | 1 | 2 | 3 | 4 | 5 |
| b. *If no:* I would find a brief screening tool useful for identification of anxiety disorders in children | 1 | 2 | 3 | 4 | 5 |
| 8. I am comfortable broaching the subject of a potential anxiety disorder with: | | | | | |
| a. the child | 1 | 2 | 3 | 4 | 5 |
| b. the family | 1 | 2 | 3 | 4 | 5 |
| 9. I believe that it is part of my responsibility in my role as a GP to identify anxiety disorders in children | 1 | 2 | 3 | 4 | 5 |
| 10. Other agencies are responsible for identifying childhood anxiety disorders | 1 | 2 | 3 | 4 | 5 |
| a. *If yes*: Name agencies here | | | | | |

| 11. To what extent do the following factors get in the way of your ability to accurately identify anxiety disorders in children: | **For the following:**<br>**1 = Not at all**<br>**2= A little**<br>**3 = Somewhat**<br>**4 = Quite a lot**<br>**5 = Very much** | | | | |
|---|---|---|---|---|---|
| a. Limitations in children's communication abilities | 1 | 2 | 3 | 4 | 5 |
| b. Misinformation from parents | 1 | 2 | 3 | 4 | 5 |
| c. My concerns about stigmatising the child | 1 | 2 | 3 | 4 | 5 |
| d. Family concerns about stigma | 1 | 2 | 3 | 4 | 5 |
| e. Cultural barriers | 1 | 2 | 3 | 4 | 5 |
| f. Language barriers | 1 | 2 | 3 | 4 | 5 |
| g. Time restrictions | 1 | 2 | 3 | 4 | 5 |
| h. Family reluctance to accept disorder | 1 | 2 | 3 | 4 | 5 |
| i. Lack of training | 1 | 2 | 3 | 4 | 5 |
| j. Lack of experience | 1 | 2 | 3 | 4 | 5 |
| k. Lack of knowledge | 1 | 2 | 3 | 4 | 5 |
| l. Lack of available/accessible treatment | 1 | 2 | 3 | 4 | 5 |
| m. Lack of effective treatment | 1 | 2 | 3 | 4 | 5 |
| Please add any further barriers to identification here if necessary | | | | | |
| n. | 1 | 2 | 3 | 4 | 5 |
| o. | 1 | 2 | 3 | 4 | 5 |
| p. | 1 | 2 | 3 | 4 | 5 |
| q. | | | | | |
| r. | | | | | |

| **Survey Item** | **Scale***<br>**1 = completely disagree**<br>**2 = disagree**<br>**3 = neutral**<br>**5 = agree**<br>**5 = completely agree**<br><br>*unless otherwise specified |
|---|---|

**Management:**
*These questions are in relation to your ongoing management of a childhood Anxiety Disorders, either prior to making a referral or in the absence of a referral*

For peer review only

| | | | | | |
|---|---|---|---|---|---|
| 12. I believe that I have had adequate training to manage anxiety disorders in children | 1 | 2 | 3 | 4 | 5 |
| 13. I have adequate skills to manage anxiety disorders in children | 1 | 2 | 3 | 4 | 5 |
| 14. I feel confident managing anxiety disorders in children | 1 | 2 | 3 | 4 | 5 |
| 15. I am comfortable discussing management strategies for anxiety disorders with: | | | | | |
| a. the child | 1 | 2 | 3 | 4 | 5 |
| b. the family | 1 | 2 | 3 | 4 | 5 |
| 16. I provide families with management tips & strategies for managing anxiety disorders in children | 1 | 2 | 3 | 4 | 5 |
| 17. I am aware of resources such as books/websites/apps to aid families in the management of anxiety disorders in children | Yes | | | No | |
| a. *If yes:* I use resources such as books/websites/apps in the management of childhood anxiety disorders | 1 | 2 | 3 | 4 | 5 |
| b. *If yes:* Please state which ones | | | | | |
| c. *If no:* I would use resources such as books/websites/apps in the management of childhood anxiety disorders | 1 | 2 | 3 | 4 | 5 |
| 18. Are you aware of any online/apps resources for parents concerned about their child's anxiety? | YES | | | NO | |
| a. *If Yes*: please state which ones | | | | | |
| b. *If Yes:* I have sign-posted parents concerned about their child's anxiety to these | 1 | 2 | 3 | 4 | 5 |
| c. *If No:* If I were aware of online/app resources, I would signpost parents to these if they were concerned about their child's anxiety | 1 | 2 | 3 | 4 | 5 |
| 19. It would be important for an online/app resource for childhood anxiety to have an evidence-base in order for me to recommend it | 1 | 2 | 3 | 4 | 5 |
| 20. It would be important to have NHS accreditation of the online/resources for childhood anxiety be in order for me to recommend it | 1 | 2 | 3 | 4 | 5 |
| 21. Having a relationship with a family aids me in the management of anxiety disorders | 1 | 2 | 3 | 4 | 5 |
| 22. I involve the child's school in the management of anxiety disorders | 1 | 2 | 3 | 4 | 5 |
| a. *If yes:* I find this helpful | 1 | 2 | 3 | 4 | 5 |
| 23. I am aware of local agencies available to support children with anxiety disorders and their families | 1 | 2 | 3 | 4 | 5 |
| 24. I believe it is part of my role as a GP to manage anxiety disorders in children | 1 | 2 | 3 | 4 | 5 |
| 25. Other agencies are responsible for managing childhood anxiety disorders | 1 | 2 | 3 | 4 | 5 |
| a. Add other agencies here if applicable | | | | | |

For peer review only

| 26. To what extent do the following factors get in the way of your ability to effectively manage anxiety disorders in children: | **For the following:**<br>**1 = Not at all**<br>**2= A little**<br>**3 = Somewhat**<br>**4 = Quite a lot**<br>**5 = Very much** | | | | |
|---|---|---|---|---|---|
| a. Cultural barriers | 1 | 2 | 3 | 4 | 5 |
| b. Language barriers | 1 | 2 | 3 | 4 | 5 |
| c. Time restrictions | 1 | 2 | 3 | 4 | 5 |
| d. Family reluctance to accept the disorder | 1 | 2 | 3 | 4 | 5 |
| e. Limitations in children's communication abilities | 1 | 2 | 3 | 4 | 5 |
| f. Misinformation from parents | 1 | 2 | 3 | 4 | 5 |
| g. My concerns about stigmatising the child | 1 | 2 | 3 | 4 | 5 |
| h. Family concerns about stigma | 1 | 2 | 3 | 4 | 5 |
| i. Lack of training | 1 | 2 | 3 | 4 | 5 |
| j. Lack of experience | 1 | 2 | 3 | 4 | 5 |
| k. Lack of knowledge | 1 | 2 | 3 | 4 | 5 |
| Please add any further barriers to management below if necessary | | | | | |
| l. | 1 | 2 | 3 | 4 | 5 |
| m. | 1 | 2 | 3 | 4 | 5 |
| n. | 1 | 2 | 3 | 4 | 5 |

For peer review

| **Survey Item** | **Scale***<br>**1 = completely disagree**<br>**2 = disagree**<br>**3 = neutral**<br>**5 = agree**<br>**5 = completely agree**<br><br>*unless otherwise specified | | | | |
|---|---|---|---|---|---|
| **Referral**<br>*These questions are in relation to a referral of a childhood Anxiety Disorder to a specialist service (such as Child & Adolescent Mental Health Services)* | | | | | |
| 27. How often in the last 5 years have you referred a child to specialist services for an anxiety disorder? | 0 times | 0-5 times | 5-10 times | 10-15 times | 15+ times |
| 28. Parental pressure increases the likelihood that I would refer a child with an anxiety disorder to specialist services | 1 | 2 | 3 | 4 | 5 |
| 29. anxiety disorders in children will generally resolve itself without treatment | 1 | 2 | 3 | 4 | 5 |
| 30. I seek specialist advice prior to making a referral of a childhood anxiety disorder | 1 | 2 | 3 | 4 | 5 |

For peer review only - http://bmjopen.bmj.com/site/about/guidelines.xhtml

| | | | | | |
|---|---|---|---|---|---|
| a. *If yes:* I find this helpful | 1 | 2 | 3 | 4 | 5 |
| b. *If no:* I think that I would find this helpful | 1 | 2 | 3 | 4 | 5 |
| 31. I experience long waiting times for referral for a childhood anxiety disorders | 1 | 2 | 3 | 4 | 5 |
| a. *If yes:* Long waiting times reduce the likelihood that I would make a referral to specialist services for childhood anxiety disorders | 1 | 2 | 3 | 4 | 5 |
| 32. I feel like I have a relationship with specialist services for childhood anxiety disorders | 1 | 2 | 3 | 4 | 5 |
| a. *If yes:* This is helpful when making a referral | 1 | 2 | 3 | 4 | 5 |
| b. *If no:* I think this would this be helpful when making a referral | 1 | 2 | 3 | 4 | 5 |
| 33. I think that specialist services' interventions for childhood anxiety disorders are likely to be effective | 1 | 2 | 3 | 4 | 5 |
| a. *If no:* This reduces the likelihood that I will make a referral | 1 | 2 | 3 | 4 | 5 |
| 34. I see my role mostly as a sign-poster/referrer for childhood anxiety disorders | 1 | 2 | 3 | 4 | 5 |
| 35. Other agencies are responsible for referring childhood anxiety disorders | 1 | 2 | 3 | 4 | 5 |
| a. *If yes:* Name other agencies here | | | | | |
| 36. To what extent do the following factors get in the way of your ability to refer children with anxiety disorders to specialist services: | **For the following:**<br>**1 = Not at all**<br>**2= A little**<br>**3 = Somewhat**<br>**4 = Quite a lot**<br>**5 = Very much** | | | | |
| a. Cultural barriers | 1 | 2 | 3 | 4 | 5 |
| b. Language barriers | 1 | 2 | 3 | 4 | 5 |
| c. Time restrictions | 1 | 2 | 3 | 4 | 5 |
| d. Family reluctance to accept the disorder | 1 | 2 | 3 | 4 | 5 |
| e. Waiting times for specialist services | 1 | 2 | 3 | 4 | 5 |
| f. Limitations in children's communication abilities | 1 | 2 | 3 | 4 | 5 |
| g. Misinformation from parents | 1 | 2 | 3 | 4 | 5 |
| h. My concerns about stigmatising the child | 1 | 2 | 3 | 4 | 5 |
| i. Family concerns about stigma | 1 | 2 | 3 | 4 | 5 |
| j. Lack of training | 1 | 2 | 3 | 4 | 5 |
| k. Lack of experience | 1 | 2 | 3 | 4 | 5 |
| l. Lack of knowledge | 1 | 2 | 3 | 4 | 5 |
| m. Lack of available/accessible treatment | 1 | 2 | 3 | 4 | 5 |

For peer review only

| | | | | | |
|---|---|---|---|---|---|
| n. Lack of effective treatment | 1 | 2 | 3 | 4 | 5 |
| Please add any further barriers to referral below if necessary | | | | | |
| o. | 1 | 2 | 3 | 4 | 5 |
| p. | 1 | 2 | 3 | 4 | 5 |
| q. | 1 | 2 | 3 | 4 | 5 |
| Please use this box to add any additional comments you may have | | | | | |

For peer review only

**STROBE 2007 (v4) Statement—Checklist of items that should be included in reports of *cross-sectional studies***

| Section/Topic | Item # | Recommendation | Reported on page # |
|---|---|---|---|
| **Title and abstract** | 1 | (*a*) Indicate the study's design with a commonly used term in the title or the abstract | 1 |
| | | (*b*) Provide in the abstract an informative and balanced summary of what was done and what was found | 2 |
| **Introduction** | | | |
| Background/rationale | 2 | Explain the scientific background and rationale for the investigation being reported | 4-5 |
| Objectives | 3 | State specific objectives, including any prespecified hypotheses | 4-5 |
| **Methods** | | | |
| Study design | 4 | Present key elements of study design early in the paper | 6-7 |
| Setting | 5 | Describe the setting, locations, and relevant dates, including periods of recruitment, exposure, follow-up, and data collection | 6-7 |
| Participants | 6 | (*a*) Give the eligibility criteria, and the sources and methods of selection of participants | 6-7 |
| Variables | 7 | Clearly define all outcomes, exposures, predictors, potential confounders, and effect modifiers. Give diagnostic criteria, if applicable | 6-7 |
| Data sources/ measurement | 8* | For each variable of interest, give sources of data and details of methods of assessment (measurement). Describe comparability of assessment methods if there is more than one group | 6-7 |
| Bias | 9 | Describe any efforts to address potential sources of bias | |
| Study size | 10 | Explain how the study size was arrived at | 6 |
| Quantitative variables | 11 | Explain how quantitative variables were handled in the analyses. If applicable, describe which groupings were chosen and why | 6-7 |
| Statistical methods | 12 | (*a*) Describe all statistical methods, including those used to control for confounding | 6-7 |
| | | (*b*) Describe any methods used to examine subgroups and interactions | 6-7 |
| | | (*c*) Explain how missing data were addressed | 6-7 |
| | | (*d*) If applicable, describe analytical methods taking account of sampling strategy | 6-7 |
| | | (*e*) Describe any sensitivity analyses | |
| **Results** | | | |

For peer review only

| | | | |
|---|---|---|---|
| Participants | 13* | (a) Report numbers of individuals at each stage of study—eg numbers potentially eligible, examined for eligibility, confirmed eligible, included in the study, completing follow-up, and analysed | 8 |
| | | (b) Give reasons for non-participation at each stage | N/a |
| | | (c) Consider use of a flow diagram | N/a |
| Descriptive data | 14* | (a) Give characteristics of study participants (eg demographic, clinical, social) and information on exposures and potential confounders | 8 |
| | | (b) Indicate number of participants with missing data for each variable of interest | 8 |
| Outcome data | 15* | Report numbers of outcome events or summary measures | 8 |
| Main results | 16 | (*a*) Give unadjusted estimates and, if applicable, confounder-adjusted estimates and their precision (eg, 95% confidence interval). Make clear which confounders were adjusted for and why they were included | 8 |
| | | (*b*) Report category boundaries when continuous variables were categorized | N/A |
| | | (*c*) If relevant, consider translating estimates of relative risk into absolute risk for a meaningful time period | N/A |
| Other analyses | 17 | Report other analyses done—eg analyses of subgroups and interactions, and sensitivity analyses | 8 |
| **Discussion** | | | |
| Key results | 18 | Summarise key results with reference to study objectives | 9 |
| Limitations | 19 | Discuss limitations of the study, taking into account sources of potential bias or imprecision. Discuss both direction and magnitude of any potential bias | 9 |
| Interpretation | 20 | Give a cautious overall interpretation of results considering objectives, limitations, multiplicity of analyses, results from similar studies, and other relevant evidence | 10 |
| Generalisability | 21 | Discuss the generalisability (external validity) of the study results | 10-11 |
| **Other information** | | | |
| Funding | 22 | Give the source of funding and the role of the funders for the present study and, if applicable, for the original study on which the present article is based | 2 |

*Give information separately for cases and controls in case-control studies and, if applicable, for exposed and unexposed groups in cohort and cross-sectional studies.

**Note:** An Explanation and Elaboration article discusses each checklist item and gives methodological background and published examples of transparent reporting. The STROBE checklist is best used in conjunction with this article (freely available on the Web sites of PLoS Medicine at http://www.plosmedicine.org/, Annals of Internal Medicine at http://www.annals.org/, and Epidemiology at http://www.epidem.com/). Information on the STROBE Initiative is available at www.strobe-statement.org.

For peer review only
